# Spectral Tuning and Photoisomerization Efficiency in Push–Pull Azobenzenes: Designing Principles

**DOI:** 10.1021/acs.jpca.0c08672

**Published:** 2020-11-10

**Authors:** Flavia Aleotti, Artur Nenov, Luca Salvigni, Matteo Bonfanti, Mohsen M. El-Tahawy, Andrea Giunchi, Marziogiuseppe Gentile, Claudia Spallacci, Alessia Ventimiglia, Giuseppe Cirillo, Lorenzo Montali, Stefano Scurti, Marco Garavelli, Irene Conti

**Affiliations:** † Dipartimento di Chimica Industriale “Toso Montanari”, Università di Bologna, Viale del Risorgimento 4, 40136 Bologna, Italy; ‡ Chemistry Department, Faculty of Science, Damanhour University, 22511 Damanhour, Egypt; § Dipartimento di Chimica “Giacomo Ciamician”, Università di Bologna, Via Selmi 2, 40126 Bologna, Italy

## Abstract

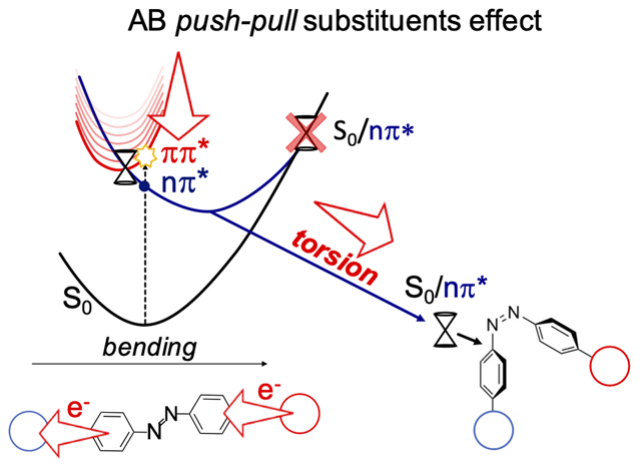

This work demonstrates how *push*–*pull* substitution can induce spectral tuning toward the visible range and improve the photoisomerization efficiency of azobenzene-based photoswitches, making them good candidates for technological and biological applications. The red-shifted bright ππ* state (S_2_) behaves like the lower and more productive dark nπ* (S_1_) state because less potential energy along the planar bending mode is available to reach higher energy unproductive nπ*/S_0_ crossing regions, which are responsible for the lower quantum yield of the parent compound. The stabilization of the bright ππ* state and the consequent increase in isomerization efficiency may be regulated *via* the strength of *push*–*pull* substituents. Finally, the torsional mechanism is recognized here as the unique productive route because structures with bending values attributable to the inversion mechanism were never detected, out of the 280 ππ* time-dependent density functional theory (RASPT2-validated) dynamics simulations.

## Introduction

**Figure 1:**
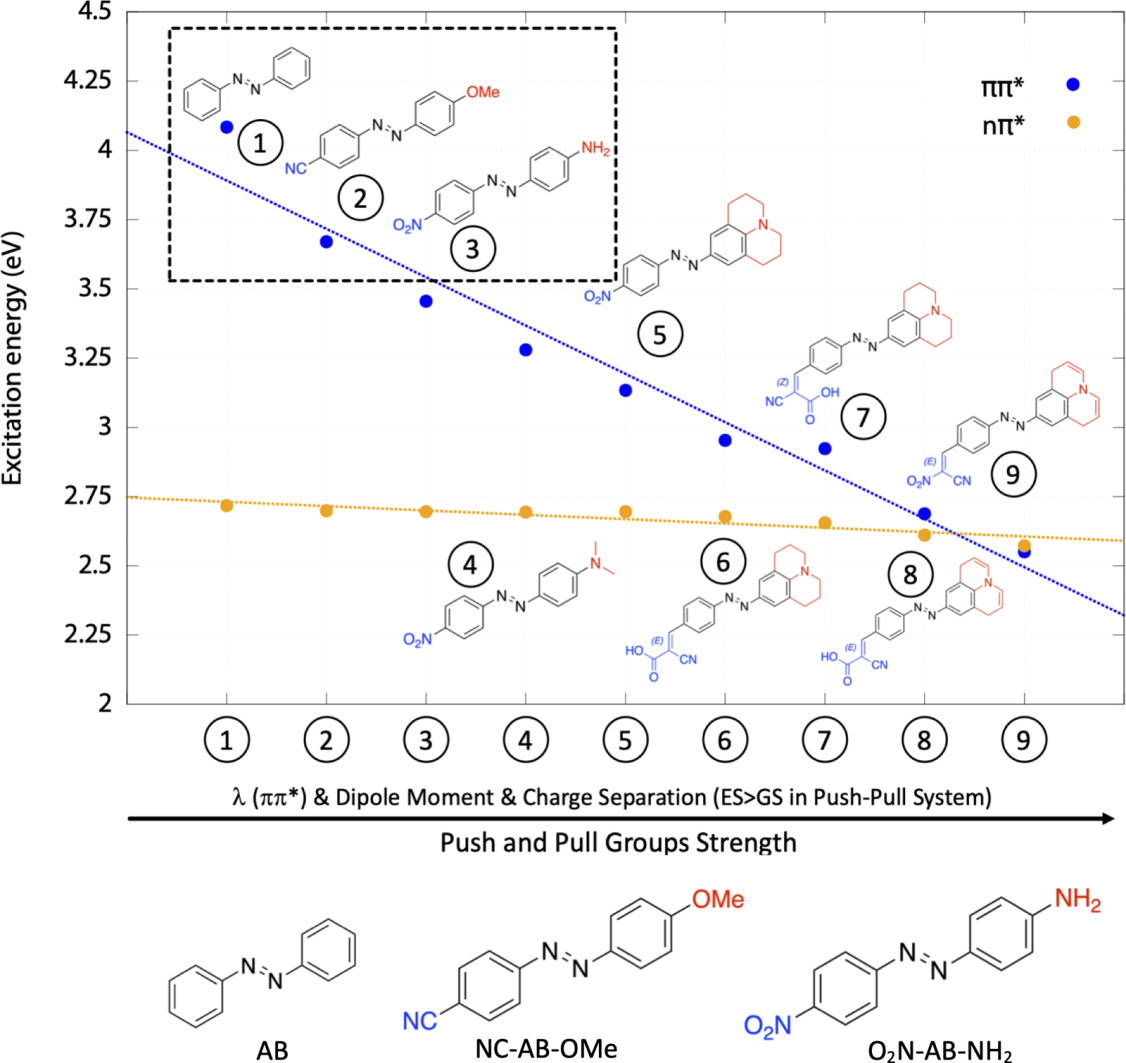
Selected AB-systems (bottom) considering an ensemble of eight *push*–*pull* derivatives: correlation between the strength of *push*–*pull* substituents and the lowest nπ*/ππ* vertical excitation energies (yellow/blue lines, respectively).

**Scheme 1:**
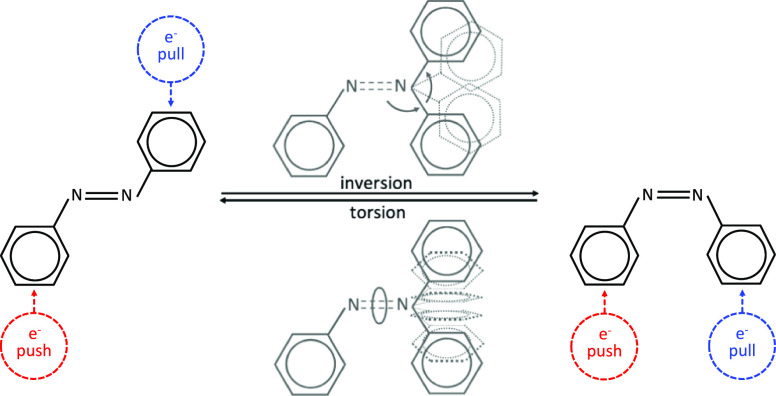
Possible Isomerization Mechanisms

Azobenzene (AB) is a prototypical photoresponsive molecule undergoing a reversible photoinduced isomerization between its *cis* and *trans* configurations, which is strongly attractive for a widespread range of applications. The *trans* ↔ *cis* interconversion mechanism has been debated for a long time:^1−10^ it could take place through rotation around the central double bond (*torsion*) or through an in-plane *bending* motion (Scheme 1). Eventually, hybrid *torsion*-*bending* processes were recently proposed.^11−14^ Interestingly, the well-separated absorption wavelengths of the two isomers make this molecule suitable for optical switches in technological^15−17^ or biological^18−20^ devices and in the development of light-powered molecular machines.^2,3,21−27^ Both isomers show two absorption bands in the UV–vis window: the more intense one is associated to a π → π* transition, peaking in the UV region (301/265 nm in the gas phase, *trans*/*cis*, respectively^28^), while the much weaker band in the visible range (440/425 nm^28^) is associated to a symmetry-forbidden n → π* transition. These ππ*/nπ* bands are separated enough to allow their selective irradiation: interestingly, excitation in the UV (ππ*) and in the visible region (nπ*) shows significantly different quantum yields (QYs), about 11% and 25%, respectively, in the *trans* case and 27% and 56% in the *cis* case in *n*-hexane.^29^ The QY wavelength dependence, which is in contrast with Kasha’s rule, suggests that different reaction mechanisms may take place starting from the ππ* or nπ* excited states (ESs),^12^ an issue that is still under discussion in experimental^8,30^ and theoretical^8,11,31−33^ studies. Because of the reversibility of the isomerization, its speed, and the simplicity of incorporating azobenzene in complex structures, many studies are focused on red-shifting the intense ππ* bands, whose UV absorption is limiting technological and biological applications. For this purpose, *push*–*pull* substituents have demonstrated to be good candidates:^37−40^ the simultaneous destabilization of the last π orbital (electron-donating substituent) and stabilization of the π* LUMO (electron-withdrawing substituent), results in red shift of the ππ* absorption,^8,18,33,41−46^ which influences the ππ–nπ* energy gap and leads to a change in the photoisomerization properties. The aim of this work is to evaluate how *push*–*pull* substituents could control the capability of AB-based photoswitches, tuning the linear absorption energy and the isomerization efficiency, depending on the mechanism behind. For this purpose, we compare the behavior of the parent AB with two different *push*–*pull*-substituted systems with increasing electron-donating/withdrawing strength: 4-methoxy-4′-cyanoazobenzene (NC–AB–OMe) and 4-(4-nitrophenylazo)aniline (O_2_N–AB–NH_2_, also known as Disperse Orange 3 or DO3); see Figure 1. The comparison is made by means of time-dependent density functional theory (TD-DFT) semiclassical dynamics simulations (RASPT2-validated at crossing points, see Table S11) accounting for multireference dynamically correlated energies. The results allow us to identify the *control knobs* of productive (*i.e.*, photoisomerization) *versus* nonproductive (*i.e.*, aborted photoisomerization) radiationless decays, thus paving the way to a rational design of AB derivatives with tuneable spectral properties and increased photoisomerization efficiency.

## Results and Discussion

Comparison between AB and the two *push*–*pull*-substituted systems NC–AB–OMe and O_2_N–AB–NH_2_ (Figure 1) was done by conducting mixed quantum–classical dynamics simulations at the TD-DFT/CAM-B3LYP/6-31G* level, following 40 gas-phase trajectories on both the *cis* and *trans* isomer of the three systems (240 trajectories in all), initiated on the bright ππ* state. Nonadiabatic events were treated with a simplified hopping scheme, relying on the energy gap as a criterion for changing the electronic state, fixed lower than 3 kcal/mol. Back hopping was always allowed between ESs, while it was not permitted once the trajectory decayed on the ground state (GS). We ran 40 additional trajectories on the *trans*-AB nπ* state that were employed as a reference for the photobehavior of the more productive dark state in the parent compound. TD-DFT/CAM-B3LYP/6-31G* was validated by benchmarking against RASPT2 static calculations at the S_1_/S_0_ crossing points, using an accurate setup that was previously tested for the parent system (MS-RASPT2/RASSCF/ANO-L-VDZP),^12,47^ where the active space (including the valence π-orbitals and the nitrogen lone pairs) was appropriately enlarged for the *push*–*pull*-substituted systems (Figures S1–S3 in the Supporting Information). However, because TD-DFT fails to produce correctly shaped potential energy surfaces (PESs) in the region surrounding intersections with S_0_, we limit our analysis to the ES dynamics until the S_1_/S_0_ gap is lower than 3 kcal/mol (S_1_/S_0_ crossing seam). The O_2_N–AB–NH_2_ and NC–AB–OMe derivatives were selected after a preliminary study (at the CAM-B3LYP/6-31G* level) of eight systems with increasing *push*–*pull* strength: Figure 1 clearly shows how the substituents red-shift the ππ* state, leaving the dark nπ* roughly unchanged. Increasing the *push*–*pull* strength reduces the ππ*/nπ* energy gap, until inversion of the ππ*/nπ* energy order (Figure 1 and Table S1). Because of their small size, the selected systems are good candidates to make accurate predictions about these promising *push*–*pull* derivatives. The quantitative accuracy of the employed method is supported by the good agreement between the experimental and computed vertical energies for *trans*/*cis*-AB, *trans*-NC–AB–OMe, and *trans*-O_2_N–AB–NH_2_ (see Table 1). This also validates the prediction for the absorption values (ππ* and nπ*), which are not available in the literature, in particular for the *push*–*pullcis*-conformers, which are thermally unstable and therefore difficult to isolate and characterize.^40^ Besides stabilizing the ππ* state, the growing strength of the *push*–*pull* substituents also affects the charge distribution on the two phenyl rings and, consequently, the molecular dipole moment (see Figure S4 and Table S1 in the Supporting Information). The charge separation is proportional to the electron-donating/withdrawing strength, as shown in Figure 1: −NH_2_ is a better *push* group than −OMe because of the lower electronegativity of the nitrogen atom; at the same time, the −NO_2_ substituent “*pulls*” more than the −CN. The charge excess on the two halves is notably larger on the bright ππ* ES, with a consequent increase in the dipole moment value: 0.162/0.247 D and 0.241/0.327 D on ππ* for *trans*/*cis* NC–AB–OMe and the O_2_N–AB–NH_2_, respectively, compared with 0.074/0.071 D and 0.115/0.106 D of the GS. The larger dipole moment of the *cis* conformer could be referred to the nonplanar geometry that hinders the orbital delocalization, leading to a larger charge separation between the two halves.

**Table 1:** Experimental, TD-DFT/CAM-B3LYP/6-31G*, and MS-RASPT2/SA-8-RASSCF/ANO-L-VDZP Vertical Excitation Energies (Oscillator Strengths in Parentheses) and Excited-State Lifetimes (τ) of *trans*- and *cis*-AB, NC–AB–OMe, and O_2_N–AB–NH_2_ in the Gas Phase^a^

		excitation energy ππ*				excitation energy nπ*					
		*trans*		*cis*		*trans*		*cis*		τ (fs)	
		nm	eV	nm	eV	nm	eV	nm	eV	*trans*	*cis*
AB	exp. value	301^28^	4.12	265^28^	4.68	440^28^	2.82	425^28^	2.92	170, 420^5^	200^b^^,^^34^
	TD-DFT	304	4.08 (0.82)	265	4.69 (0.18)	456	2.72 (0.00)	464	2.67 (0.03)	168, 231, 323 (tors. path)	242, 278
	RASPT2	322	3.85 (0.42)	302	4.11 (0.05)	478	2.59 (0.00)	450	2.75 (0.02)	−	−
NC–AB–OMe	exp. value^c^	380^35^	3.26	−		460^35^	2.70	−		−	−
	TD-DFT	338	3.67 (1.10)	288	4.30 (0.40)	459	2.70 (0.00)	472	2.62 (0.05)	70, 225, 386 (tors. path)	181, 221
	RASPT2	342	3.62 (0.60)	322	3.85 (0.15)	509	2.44 (0.00)	474	2.62 (0.05)	−	−
O_2_N–AB–NH_2_	exp. value	353^36^	3.51	−		442^36^	2.81	−		−	−
	TD-DFT	359	3.46 (1.10)	313	3.96 (0.35)	460	2.70 (0.00)	472	2.62 (0.07)	86, 227, 300 (tors. path)	118, 144
	RASPT2	414	2.99 (0.84)	348	3.56 (0.15)	453	2.74 (0.00)	506	2.45 (0.05)	−	−

a Optimized GS *bending* and *torsional* parameters are shown at the top of Figures 2 and 4 (Cartesian coordinates for the *trans* and *cis* conformers are given in the Supporting Information). Details on the S_2_ and S_1_ average lifetimes, calculated on all trajectories or separately on the *torsional* or *bending* paths, are documented in Tables S4–S6, respectively.

b In ethanol at room temperature.

c In 2-methyltetrahydrofuran (MTHF) at 77 K.

We show how the *push*–*pull* derivatives behave dynamically different, compared to the parent system, when they are excited to the bright ππ* state in the following. Because photoexcited *trans*- and *cis*-isomers lead to quite different paths^11^ (as demonstrated by the experimental lifetimes in Table 1), we will discuss them separately.

### *trans*-AB Systems

Looking at the S_2_ dynamics leading to the initial S_2_ → S_1_ decay, we notice that the CNNC dihedral angle stays close to 180° in all the systems, while the CNN bending angles close and then oscillate around a value that is a bit smaller than that in the FC geometry, in agreement with recent studies on the AB photoisomerization.^12^Figure 2 shows the normalized distribution of the CNNC torsion (top) and CNN bending (bottom) trajectories along the *trans*-dynamics of the three systems, including the nπ* *trans*-dynamics of AB. The left panels refer to the dynamics on S_2_ [panels (a–c) and (h–j)], while the right plots refer to the dynamics on S_1_ after decay from S_2_ [panels (e–g) and (l–n)] or after direct excitation [for *trans*-AB, panels (d,k)]. The most significant effect of *push*–*pull* substitution is a drastically shorter ππ* lifetime with respect to the parent compound, where S_2_ is living two times longer than in the substituted *trans*-systems (168 fs for AB against 70 and 86 fs for NC–AB–OMe and O_2_N–AB–NH_2_, respectively; see vertical dashed lines in Figure 2, left part).

**Figure 2:**
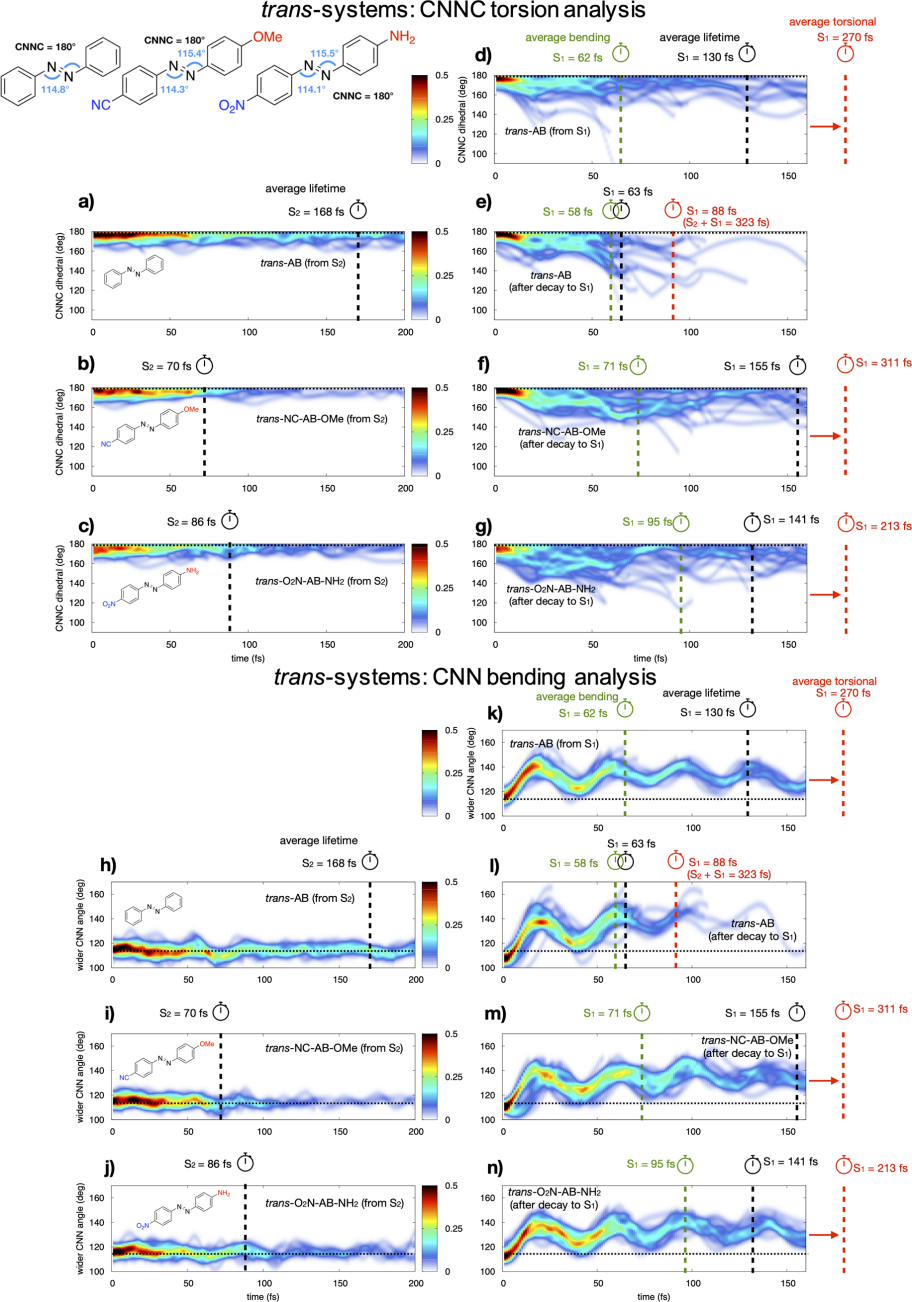
Normalized distribution of the CNNC *torsional* value (top panels) and widest CNN bending value (bottom panels) over time for the *trans*-system dynamics (40 for each panel) on S_2_ (left) and on S_1_ (right) until decay to the GS. The color scale refers to the normalized density of trajectories. The panels (d,k) refer to the 40 dynamics initiated in the nπ* for *trans*-AB. Vertical dashed lines: ES lifetimes averaged over all trajectories (black) and over *torsional* (red) or *bending paths* (green). Horizontal dotted lines: FC value of the relative coordinate. Top left structures: CNNC torsion and CNN bending values in the S_0_ minimum *trans*-systems (DFT/B3LYP/6-31G* optimized).

On the other hand, in the subsequent dynamics on the nπ* state (S_1_), bending oscillations accompany the torsional motion (Figure 2, right part), leading to a S_1_ → S_0_ crossing region spanning from planar to fully rotated CNNC values (Tables S4–S6 in the Supporting Information). This is due to an extended S_0_/S_1_ crossing seam, that has been extensively documented in previous studies,^11,47,48^ covering both *bending* and *torsional* modes, where the fully (∼90°) rotated structures are the lowest in energy, but also, higher energy, less-rotated structures could be accessible through the bending mode, provided that enough kinetic energy is available in the dynamics. Based on the characteristics of the S_1_ → S_0_ seam, we have grouped the trajectories in two different sets, labelled *torsional* and *bending paths*, based on the CNNC torsional value at the S_1_ → S_0_ decay: the former includes trajectories decaying on S_0_ at CNNC < 135° (half between 180 and 90°), the latter includes trajectories which, to a great extent, preserve the planarity of azobenzene until decaying to the GS (CNNC > 135°). Most trajectories for all the three *trans*-systems follow the *bending path* (82.5/65/65% for AB/NC–AB–OMe/O_2_N–AB–NH_2_, respectively; see Table 2), but none of them reach bending values that could justify a possible *inversion*-driven isomerization process (*i.e.*, close to 180°; see Scheme 1, bottom part of Figure 2, and Tables S4–S6). This explains the smaller QY of *trans*-AB from ππ* (11% vs 25% from nπ*^29^): the most populated bending paths are reaching S_0_/S_1_ CIs with neither *bending* nor *torsion* values large enough to allow the *trans*–*cis* isomerization. Moreover, the *bending* motions are mainly symmetric (see values in Tables S4–S6), and even a hypothetical concerted bending mechanism would lead back to the reagent. Consequently, on the basis of the large number of dynamics on the three *trans*-systems (120, 40 for each system), we conclude that the only productive process follows the *torsion* mechanism. However, because our analysis is limited to the ES dynamics until decay to the GS, we can only have an upper bound estimate of the ππ* QY, which is given by the number of *torsional paths* populated for each system: we obtained 17.5%, 35%, and 35% for *trans*-AB, NC–AB–OMe, and O_2_N–AB–NH_2_, respectively, envisaging a larger QY in the *push*–*pull* systems than that in the parent AB (see Table 2). To prove that the isomerization QY correlates well with the population of the *torsion* mode, we ran 40 dynamics for *trans*-AB (using the same initial conditions as for the ππ* state) starting from the more productive nπ* state (experimental QY = 25%^29^): in this case, 32.4% of the trajectories belong to the *torsional* path (Table 2), a value that is close to the observed QY. Previous semiclassical dynamics by Granucci and Persico^49^ employing a semiempirical Hamiltonian reported values for the QYs of 15% and 33% starting from the ππ* and nπ*state, respectively, which is perfectly in line with the amount of torsional trajectories obtained in each case from our simulations. Remarkably, the ratio between the *torsional paths* populated when initiating the dynamics either in the ππ* or nπ* state (Table 2) matches well with the experimental ππ* and nπ* QY ratio (theoretical estimate: 17.5/32.4 = 0.54, experimental QY ratio in *n*-hexane:^29^ 11/25 = 0.44). This strengthens the hypothesis that CN=NC torsion is the productive mechanism, which explains the larger QY in the *transpush*–*pull* systems.

**Table 2:** Analysis of the Decay geometries^a^

		*torsional* path				*bending* path			
		AB		NC–AB–OMe	O_2_N–AB–NH_2_	AB		NC–AB–OMe	O_2_N–AB–NH_2_
		ππ*	nπ*	ππ*	ππ*	ππ*	nπ*	ππ*	ππ*
*Trans*-Systems									
	relative amount (%)	17.5	32.4	35.0	35.0	82.5	67.6	65.0	65.0
S_2_ → S_1_ hop	CNNC (deg)	173		177	173	175		176	173
	CNN–NNC (deg)	108–105		108–111	114–110	108–104		107–110	110–113
	N=N (Å)	1.40		1.39	1.32	1.40		1.36	1.32
S_1_ → S_0_ hop	CNNC (deg)	126	119	123	123	157	156	161	158
	CNN–NNC (deg)	145–141	139–134	130–136	138–131	149–142	147–141	147–142	144–139
	N=N (Å)	1.24	1.24	1.28	1.28	1.24	1.23	1.23	1.23
*Cis*-Systems									
S_2_ → S_1_ hop	CNNC (deg)	12		14	14				
	CNN–NNC (deg)	127–113		131–112	131–120				
	N=N (Å)	1.43		1.39	1.26				
S_1_ → S_0_ hop	CNNC (deg)	79		74	75				
	CNN–NNC (deg)	132–111		138–115	128–115				
	N=N (Å)	1.31		1.31	1.29				

a *Trans*-system dynamics: torsional path = CNNC < 135° at the S_1_/S_0_ decay and bending path = 135° < CNNC < 180° at the S_1_/S_0_ decay. The geometrical parameters are averaged over all the set of trajectories belonging to each *torsional*/*bending* group. *Cis*-system dynamics: all trajectories are ascribable to the *torsional* path (>99%), for which CNNC > 45° at the S_1_/S_0_ decay.

**Figure 3:**
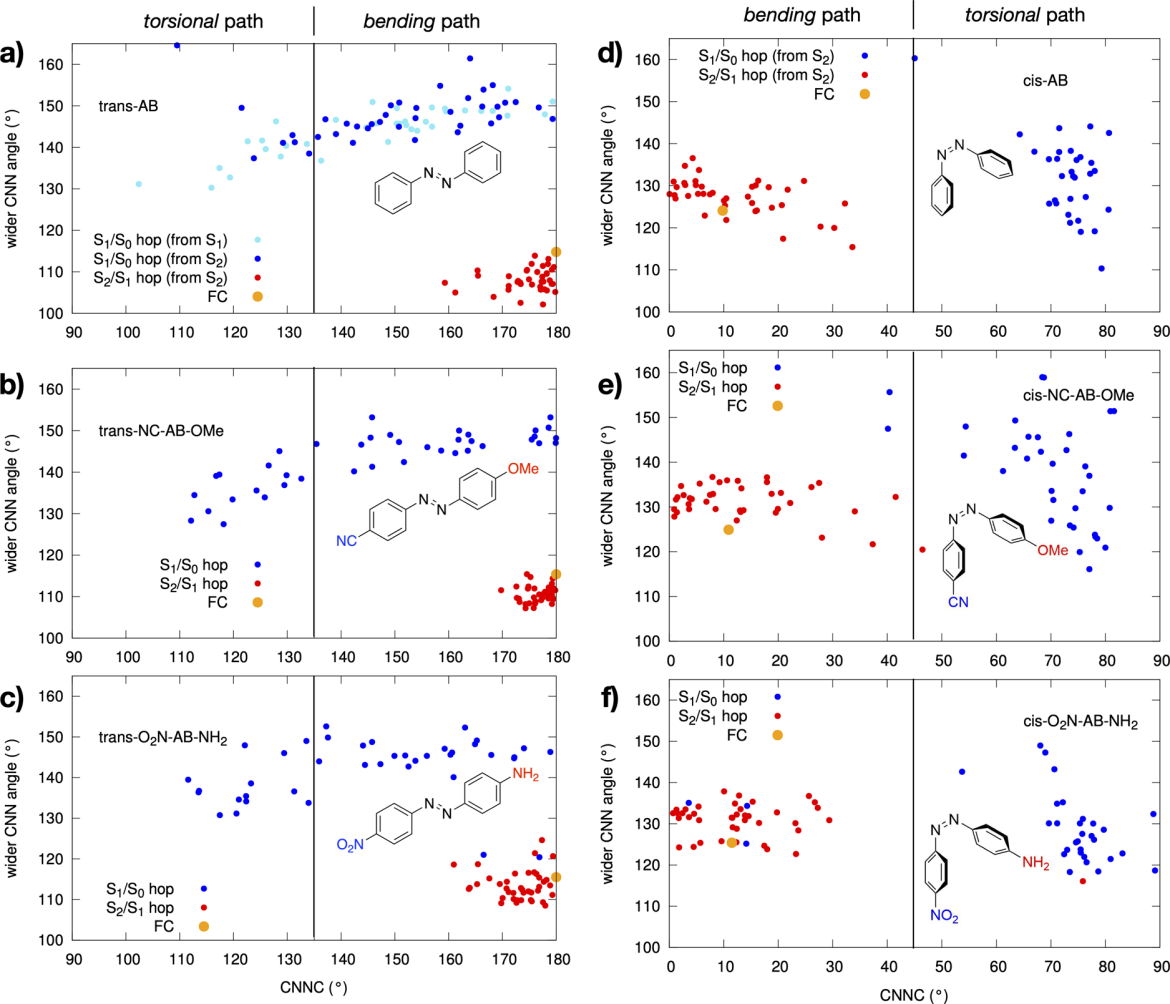
Projection of all the decay geometries in the *torsion*/*bending* space for the *trans* (left part) and *cis* (right part) dynamics. Red points = S_2_ → S_1_, blue points = S_1_ → S_0_ hopping point distribution populated along the ππ* (S_2_) dynamics of the three systems. Light-blue points in panel (a) correspond to S_1_ → S_0_ hopping points populated by the 40 trajectories starting from the *trans*-AB nπ* (S_1_) state. The vertical line in each panel defines the *torsional* and the *bending* regions (i.e., half way between 180 and 90° for the *trans*-isomers and between 0 and 90° for the *cis* ones).

To further support and rationalize that the *push*–*pull* systems could be more productive than the parent *trans*-AB because of the larger population of *torsional paths*, Figure 3 shows the S_2_ → S_1_ (red) and the S_1_ → S_0_ (dark blue) hopping point distribution, along the *bending*/*torsional* coordinates, for the ππ* trajectories of the three different systems. Interestingly, for the *push*–*pull* systems, the S_1_ → S_0_ hopping point distribution obtained starting from the bright ππ* state is matching with the *trans*-AB distribution obtained starting from the more efficient nπ* state [light-blue points in Figure 3 panel (a) *versus* blue points in panels (b,c)], envisaging that the *push*–*pull* derivatives excited to ππ* behave exactly as AB excited to nπ*, for which a larger isomerization productivity is experimentally documented. Instead, the S_1_ → S_0_ decay points for *trans*-AB when excited to S_2_ show a clearly different distribution, largely concentrated in the *bending* region. Additionally, the average bending values at the S_1_ → S_0_ hopping points are a bit smaller for the *torsional* trajectories (and in the nπ* dynamics) than for the *bending* ones (see Table 2 and Figure 3), which is perfectly in line with the shape of the S_1_/S_0_ crossing region depicted in our earlier studies,^11^ showing that fully rotated CIs (∼90°) display smaller bending values than less-rotated (and therefore less productive) ones.^11,47^ It is thus apparent that by calibrating the strength of *push*–*pull* substituents, one could red-shift the absorption maximum of the bright ππ* state, bringing it closer to that of the productive nπ* and concurrently increase the photoisomerization efficiency, two main achievements in the design of photoactive AB-based systems.

Concerning the lifetimes, we see a nice agreement between experiments and theory: time-resolved photoelectron spectroscopy experiments^5^ show two decay time constants for *trans*-AB: the shorter (170 fs) is perfectly matching our *trans*-AB S_2_ → S_1_ average decay time value of 168 fs (black dashed line in Figure 2a,h and in Table 1); the longer one (420 fs) is close to the computed S_2_ + S_1_ average decay time of 323 fs of the slower *torsional paths* (red dashed line in Figure 2e,l; see also Table 1 and details on average lifetimes in Table S4). Even though the original work^5^ attributed the longer experimental lifetime of 420 fs to two higher lying ππ* states (S_3_–S_4_), the low oscillator strength reported for them^5,11^ suggests that the population of S_2_ is by far more probable and that the 420 fs time constant could instead be associated to the S_2_ + S_1_ deactivation following the CNNC torsional motion toward the twisted S_1_/S_0_ crossing region. This hypothesis was already proposed by Granucci *et al.*,^49^ and it is also supported by the following theoretical^50−52^ and experimental^34^ studies reporting a S_1_ lifetime of about 0.4 ps.

An insight into the behavior of the dynamics following S_2_ → S_1_ decay clearly shows an average nπ* S_1_ lifetime that is almost doubled in the *push*–*pull* derivatives than in AB (155 fs, 141 fs, and 63 fs for NC–AB–OMe, O_2_N–AB–NH_2_, and AB, respectively, black dashed lines in Figure 2e–g). Interestingly, the S_1_ average lifetimes of the *push*–*pull* systems resemble those of the more productive dark nπ* state of the parent AB when it is directly excited, (130 fs, see black dashed line in Figure 2d,k), once again showing that the dynamics of the *push*–*pull* systems excited to the ππ* resembles that of the nπ* state of AB. Eventually, we observe that the S_1_*torsional path* average lifetime in the *transpush*–*pull* systems (red dashed line, Figure 2f,g) is about three times longer than that in the *bending* paths (green dashed lines Figure 2f,g), which is again similar to the dynamics of *trans*-AB from the nπ* state (62 vs 270 fs, Figure 2d,k). The longer lifetime of the *torsional versus bending* path could be simply referred to the time needed for internal vibrational energy redistribution from the *bending* to *torsional* mode, which is necessary to populate the MEP leading to the nπ* decay process to the GS.^47^ This is in line with the recently published AB ππ* CASPT2 dynamics,^12^ indicating that the productive CN=NC *torsional* mechanism is slower than the unproductive route characterized by symmetric *bending* modes.

**Scheme 2:**
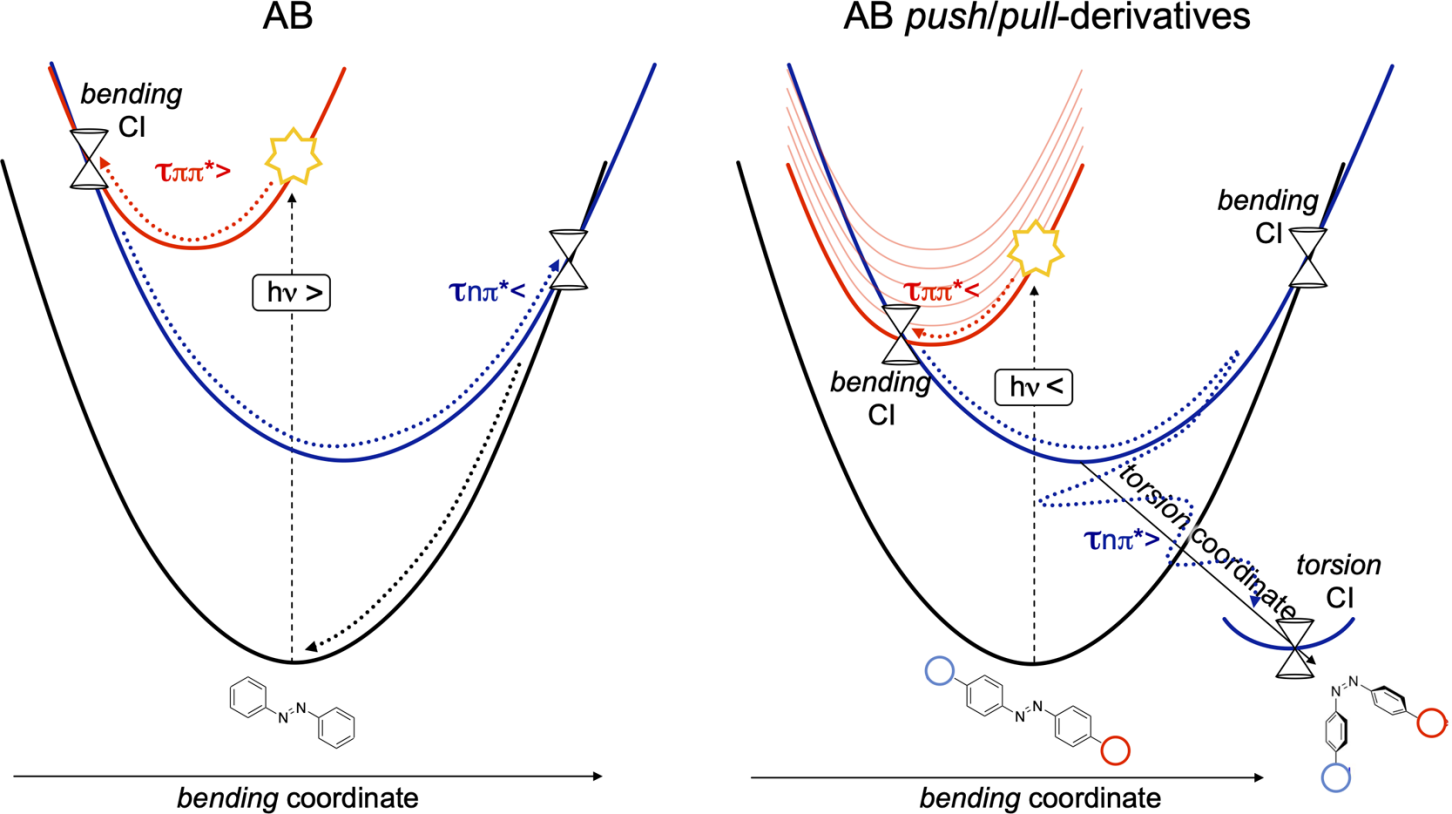
*Push*–*Pull* Substitution Effect

To explain the opposite trend in the S_2_ and S_1_ lifetimes observed in *push*–*pull* AB as compared to the parent compound, we propose a simple model, which rationalizes entirely the differences documented in both ESs for the three systems. Because the *push*–*pull* substituents stabilize only the bright state, while keeping the nπ* energy unaffected, we imagine a simple shift of the ππ* PES, as shown in Scheme 2. By lowering the ππ* state, the crossing with nπ* becomes more accessible (*i.e*., lower activation energy), thus leading to a shorter S_2_ lifetime for the *push*–*pull* derivatives (Figure 2). Additionally, less energy becomes available along the initially populated bending modes on S_1_ to eventually access the higher energy S_1_/S_0_ crossing region at roughly planar structures (torsional angle CNNC around 180°). Eventually, vibrational energy redistribution takes place, triggering population of the nπ* (*torsional*) minimum energy path and populating the slower, but more productive, torsional paths leading to rotated S_1_/S_0_ CI structures.

### *cis*-AB Systems

**Figure 4:**
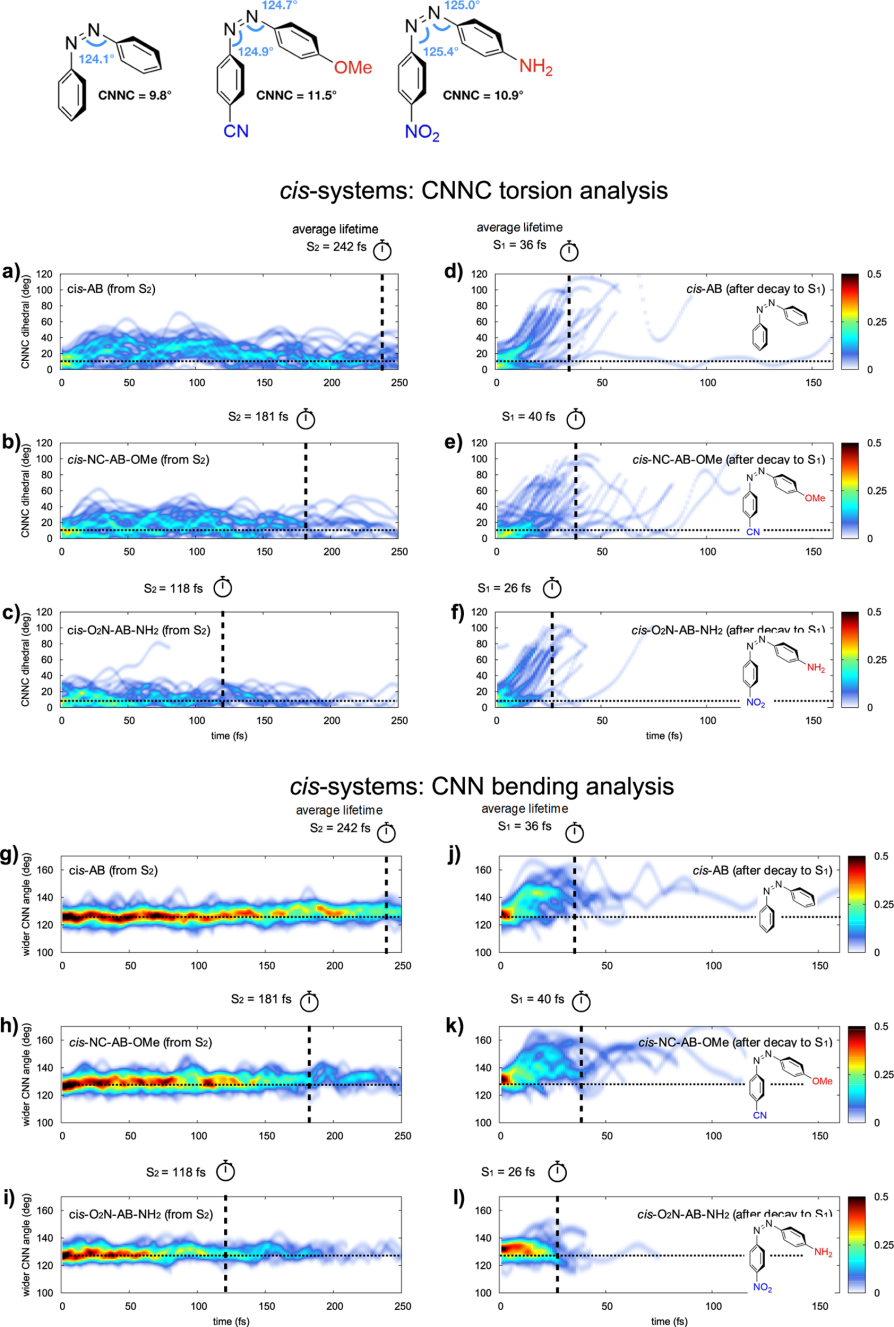
Normalized distribution of the CNNC *torsional* value (top panels) and widest CNN bending value (bottom panels) over time for the *cis*-system dynamics (40 for each panel) on S_2_ (left) and on S_1_ (right) until decay to the GS. The color scale refers to the normalized density of trajectories. Vertical dashed lines: ES lifetimes averaged over all trajectories. Horizontal dotted lines: FC value of the relative coordinate. Top left structures: CNNC torsion and CNN bending values in the S_0_ minimum *cis*-systems (DFT/B3LYP/6-31G*-optimized).

*Cis*-isomers behave similarly to the *trans* ones: the *push*–*pull* substituents red-shift the ππ* intense band according to their electron-donating/withdrawing strength, leaving the nπ* state energy roughly unchanged (Table 1). The main difference with respect to the *trans*-conformers is that except for few outliers, more than 99% of the 120 *cis*-dynamics reach S_1_ → S_0_ regions, which is always attributed to the CNNC *torsional* decay mechanism (CNNC > 45°; see Tables 2 and S7–S9 in the Supporting Information), as clearly shown in Figure 3. This is in line with the larger experimental QY observed in *cis*-AB (Φ = 0.27 vs 0.11 of the *trans*(29)). Moreover, *torsion* is activated already on S_2_ (reaching torsion values up to 50°; see Figure 3) and becomes notably larger on the S_1_, as shown by the torsion panels of Figure 4, because of the nonplanar FC starting structure. The earlier activation of the *torsional* motion, compared to the *trans* analogues, impedes the early decay to the nπ* state through the *bending* funnel, resulting in longer S_2_ lifetimes of the *cis*-isomers, compared to the *trans* ones, in agreement with previous dynamics simulations of AB from the ππ* state.^52^ The bending motions are more asymmetric than those in the *trans*-systems (Table 2), and to be more specific, the larger bendings are mainly attributed to the fragment bearing the electron donor group (−OMe or −NH_2_; see Tables S8 and S9 in the Supporting Information). Anyways, none of the *cis*-dynamics reach bending angles close to 180° (see Tables S7–S9), suggesting that the *inversion* path is not populated, as already noted for *trans*-systems. The S_2_ lifetime is shortening with the increasing *push*–*pull* strength (Figure 4a–c and g–i), supporting the previously explained hypothesis that the ππ* red shift speeds up the access to the ππ*/nπ* crossing seam (Scheme 2). Instead, the nπ* lifetime in the *cis*-isomers is not affected by the *push*–*pull* substituents (Figure 4d–f and j–l) because the steeper gradient along the torsional coordinate drives the system straight to the rotated nπ*/S_0_ peaked CIs, as documented previously.^11,53^ These differences in the S_1_ PES shape (compared to the flat *trans*-nπ* surface) correlate with a larger amount of kinetic energy along the torsional mode, inevitably leading to an increased photoisomerization QY with respect to the *trans* analogues.

That said, looking at the S_2_/S_1_ and S_1_/S_0_ CI distribution along the torsion/bending coordinates in Figure 3, we see that the parent and *push*–*pull*-derivatives behave similarly, populating the same photoisomerization processes and thus suggesting similar photoisomerization QYs (which is expected to remain higher than that in the *trans* analogues).

## Conclusions

Kasha’s rule violation in AB systems was often attributed to two different decay channels that are populated when exciting directly the ππ* (S_2_) or nπ* (S_1_) state. The present work supports and extends this hypothesis by proposing a unified mechanistic model, which can be applied to both azobenzene and its *push*–*pull* derivatives, foreseeing a higher QY for the latter, with respect to the parent compound. By analyzing a large number of TD-DFT (RASPT2 validated) ππ* molecular dynamics on AB and two *push*–*pull* AB derivatives, we see that S_2_ trajectories in the parent compound are mainly characterized by CNN/NNC bending motions preserving the system planarity and eventually leading to S_2_/S_1_ and, subsequently, to S_1_/S_0_ crossing regions, which are unproductive and drive the systems back to reactant repopulation. Indeed, only a small number of trajectories redistributes the vibrational energy along the *torsional* mode that could drive the system to the fully rotated S_1_/S_0_ CI (∼90°), triggering the isomerization. We demonstrate that *push*–*pull* substituents mitigate this situation, leading to a behavior from the ππ* state (bright) that is similar to that of the productive nπ* state (dark). Indeed, the substituents induce a ππ* red shift, bringing the bright state closer to the dark nπ* and therefore leading to the population of the same (and more productive) torsional pathways (Scheme 2). This demonstration, based on a significant number of trajectories, endorses the *push*–*pull* derivatives as flexible candidates for more efficient and visible light-activated switches, which are attractive for technological and biological applications. Moreover, the large number of trajectories is a strong statistical support to finally assign the photoisomerization process exclusively to the torsion mechanism, even if it is assisted by large CNN/NNC bending motions.^11,12,14,48,53^ Indeed, structures distorted enough to support a photoisomerization driven by the *inversion* route are never reached (Scheme 1). Therefore, only the *torsion* is the productive path, while the pure *bending* is an unproductive reaction coordinate, justifying the lower QY observed in AB when exciting the ππ* state (*bending*-dominated) as compared to direct nπ* excitation (*torsion*-driven). Because of the importance of the embedding on the excited-state dynamics,^54−56^ QM/MM studies are currently undergoing to disclose effects of solvent polarity and viscosity on the photoactivity of these systems.

## Supporting Information Available

Computational details, vertical excitation energies and dipole moments of AB *push*−*pull* derivatives, nature of TD-DFT and RASPT2 ESs, charge distribution in the FC geometry, details on TD-DFT dynamics, RASPT2 energies at S_1_/S_0_ crossings (0 K dynamics), and Cartesian coordinates of the B3LYP/DFT/6-31G*-optimized GS minima (PDF)
