# Supplementary material for: Spectral Tuning and Photoisomerization Efficiency in Push–Pull Azobenzenes: Designing Principles
Source: J Phys Chem A. 2020 Nov 10;124(46):9513–23. doi: 10.1021/acs.jpca.0c08672 (PMC8015210; doi:10.1021/acs.jpca.0c08672)
Supplement: Supplementary file 1 — jp0c08672_si_001.pdf [file jp0c08672_si_001.pdf]

Spectral Tuning and Photoisomerization Efficiency in Push-Pull Azobenzenes: Designing Principles.

Flavia Aleotti[a] ‡, Artur Nenov[a] ‡, Luca Salvigni[a], Matteo Bonfanti[a], Mohsen M. El-Tahawy[a,b], Andrea Giunchi[a], Marziogiuseppe Gentile[c], Claudia Spallacci[a], Alessia Ventimiglia[a], Giuseppe Cirillo[a], Lorenzo Montali[a], Stefano Scurti[a], Marco Garavelli*[a], Irene Conti*[a].

[a] Dipartimento di Chimica industriale "Toso Montanari", Università di Bologna, Viale del Risorigmento 4, 40136 Bologna, Italy.

[b] Chemistry Department, Faculty of Science, Damanhour University, Damanhour 22511, Egypt.

[c] Dipartimento di Chimica "Giacomo Ciamician", Università di Bologna, Via Selmi 2, 40126 Bologna, Italy.

Supporting Information

1. Computational details
2. RASSCF/RASPT2 calculations: active space orbitals
3. Vertical excitation energies and dipole moments of AB-*push-pull* derivatives
4. Excited state nature
5. Ground state and excited state charge distribution in the FC geometry
6. TD-DFT Dynamics
7. TD-DFT *vs* RASPT2 energies at S_1/S_0 crossings (0K dynamics)
8. Cartesian coordinates of the B3LYP/DFT/6-31G* optimized ground state minima

1. Computational details

For each of the six considered systems (three *cis* and three *trans*, AB-compounds), we generated 40 initial conditions sampled from a Wigner distribution at 300K using B3LYP/6-31G* normal modes and frequencies (obtained at the corresponding ground state equilibrium structure) through an interface with a stand-alone script part of the quantum molecular dynamics program JADE[1]. High frequency modes (>2000 cm^{-1}, *i.e.*, C-H stretching) were excluded from the sampling. For each frame, we ran semi-classical dynamics starting from the lowest $\pi\pi^*$ bright state (usually S_2) following Newton's equations of motion for the nuclei and calculating the electronic energy gradient at the TD-DFT/CAM-B3LYP/6-31G* level of theory, using a timestep of 1 fs and a time propagation of 1500 fs. Nonadiabatic events were treated with a simplified hopping scheme relying on the energy gap as a criterium for changing the electronic state, fixed lower than 3 kcal/mol. Back hopping was always allowed between ESs, while it was not permitted once the trajectory decayed on the ground state (GS).

For the *trans*-AB parent system, we started 40 additional dynamics simulations starting from the nπ* state (S_1) (out of which 3 were discarded due to convergence problems, resulting in 37 trajectories), using the same starting frames as for the ππ* state dynamics, in order to see the possible differences in the photoisomerization mechanism.

As an assessment for the TD-DFT data, we calculated the vertical excitation energies and the oscillator strength at the GS minimum geometry, both at the CAM-B3LYP/6-31G*/TD-DFT and at the RASPT2/RASCF/ANO-L-VDZP level of theory for each system, focusing our attention on the lowest nπ* and the lowest bright ππ* states. The accuracy of the RASPT2/RASSCF/ANO-L-VDZP protocol was previously-tested[2,3], and its results were therefore used as reference. We performed SS-8-RASPT2/SA-8-RASSCF/ANO-L-VDZP vertical calculations using different active space dimensions for the three different systems: for *trans*- and *cis*-AB (4,9|0,0|4,7), for *trans*-NC-AB-OMe (2,9|10,8|2,6), for *cis*-NC-AB-OMe (2,6|10,9|2,4), for *trans*-O_2N-AB-NH_2 (2,9|10,6|2,5) and for *cis*-O_2N-AB-NH_2 (2,9|10,7|2,4). The differences in the number of active space orbitals and/or orbital distribution between the corresponding *trans* and *cis* isomers are due to the high computational cost of the RASPT2/RASSCF calculations, which made sometimes necessary to exclude some low-contributing orbitals and/or change the distribution in RAS1, 2 and 3. The active space orbitals for each system are documented in Figure S1,Figure S2 Figure S3.

In order to assess the accuracy of the TD-DFT dynamics, we calculated, besides the Franck-Condon (FC) energies at the RASSCF/RASPT2 level, also the GS and the first ES energy at the S_1/S_0 crossing regions, in particular at the point where the system decays on the GS in the 0K TD-DFT dynamics simulations (*i.e.*, dynamics started at the GS minimum geometry, without any initial velocity), starting from the bright state (ππ*). The good matching between the high level multi reference RASPT2 method and the single reference TD-DFT (Table S1) is enforcing the reliability of the latter method, as already demonstrated previously[4].

All the dynamics simulations and the TD-DFT calculations were performed using the suite COBRAMM[5] interfaced with the software GAUSSIAN[6] for the energy calculations, while the RASSCF/RASPT2 calculations were performed using the OpenMolcas[7] quantum chemistry program.

2. RASSCF/RASPT2 calculations: active space orbitals

Trans-azobenzene

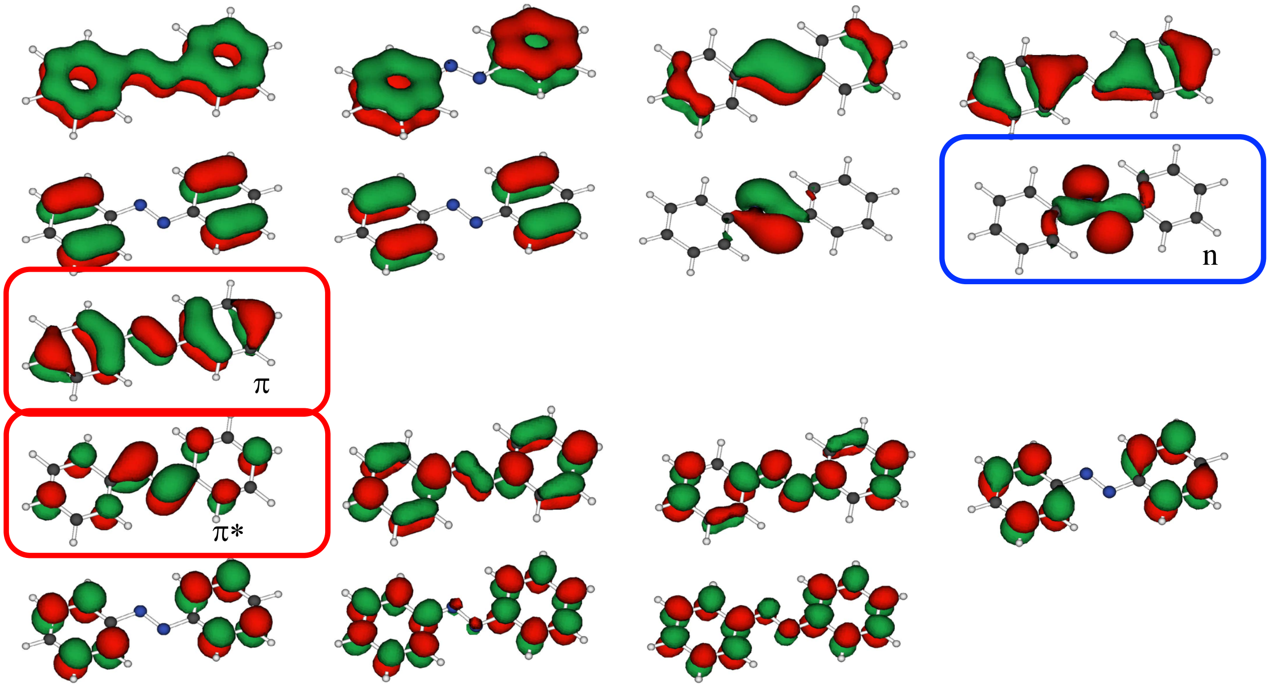

Figure S1- Active space orbitals for *trans*-azobenzene. Corresponding *cis*-orbitals were used in the case of the *cis* isomer.

Trans-4-methoxy-4'-cyanoazobenzene

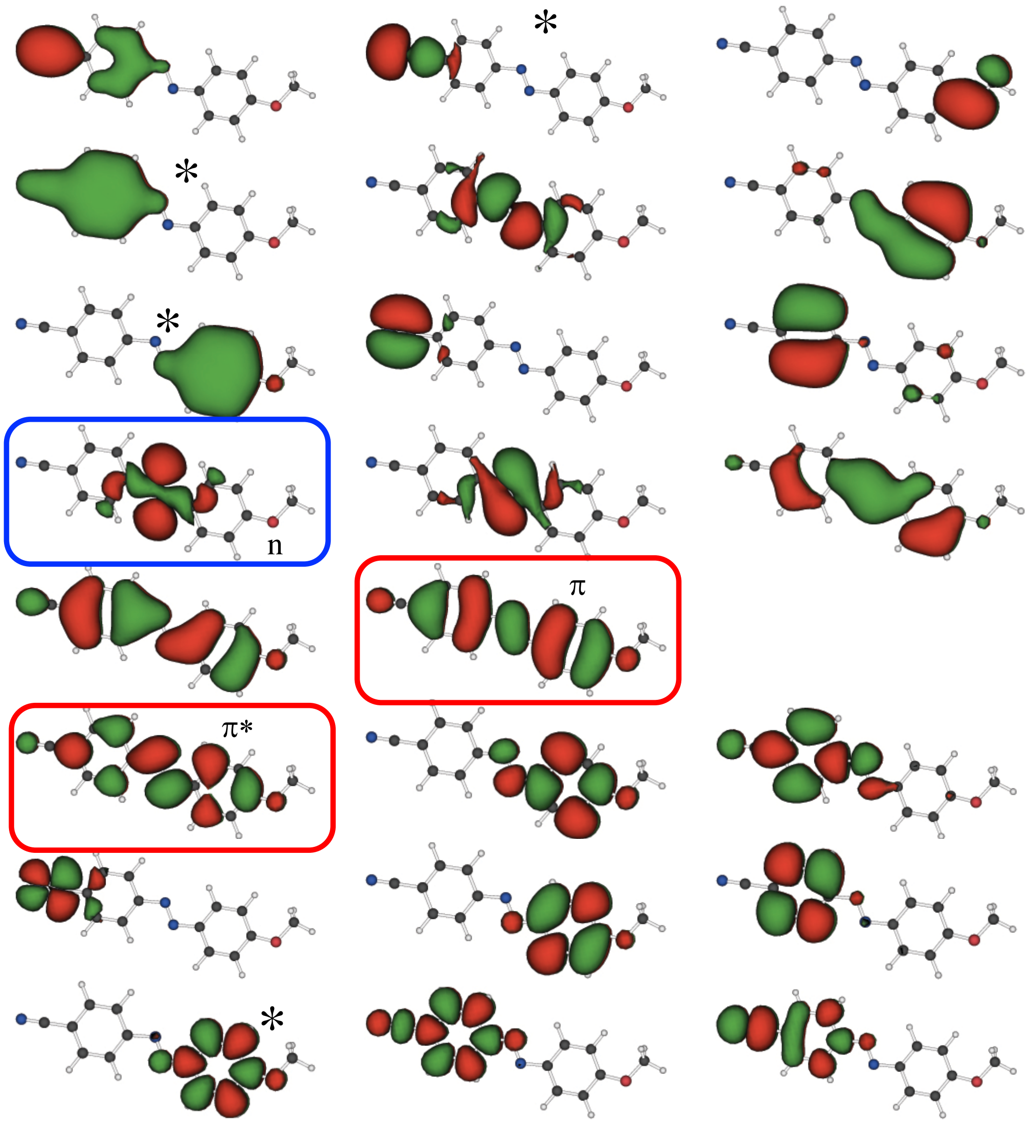

Figure S2 – Active space orbitals for *trans*-4-methoxy-4'-cyanoazobenzene. Corresponding *cis*-orbitals were used in the case of the *cis* isomer, excluding the ones marked with *.

Trans-4-(4-Nitrophenylazo)aniline

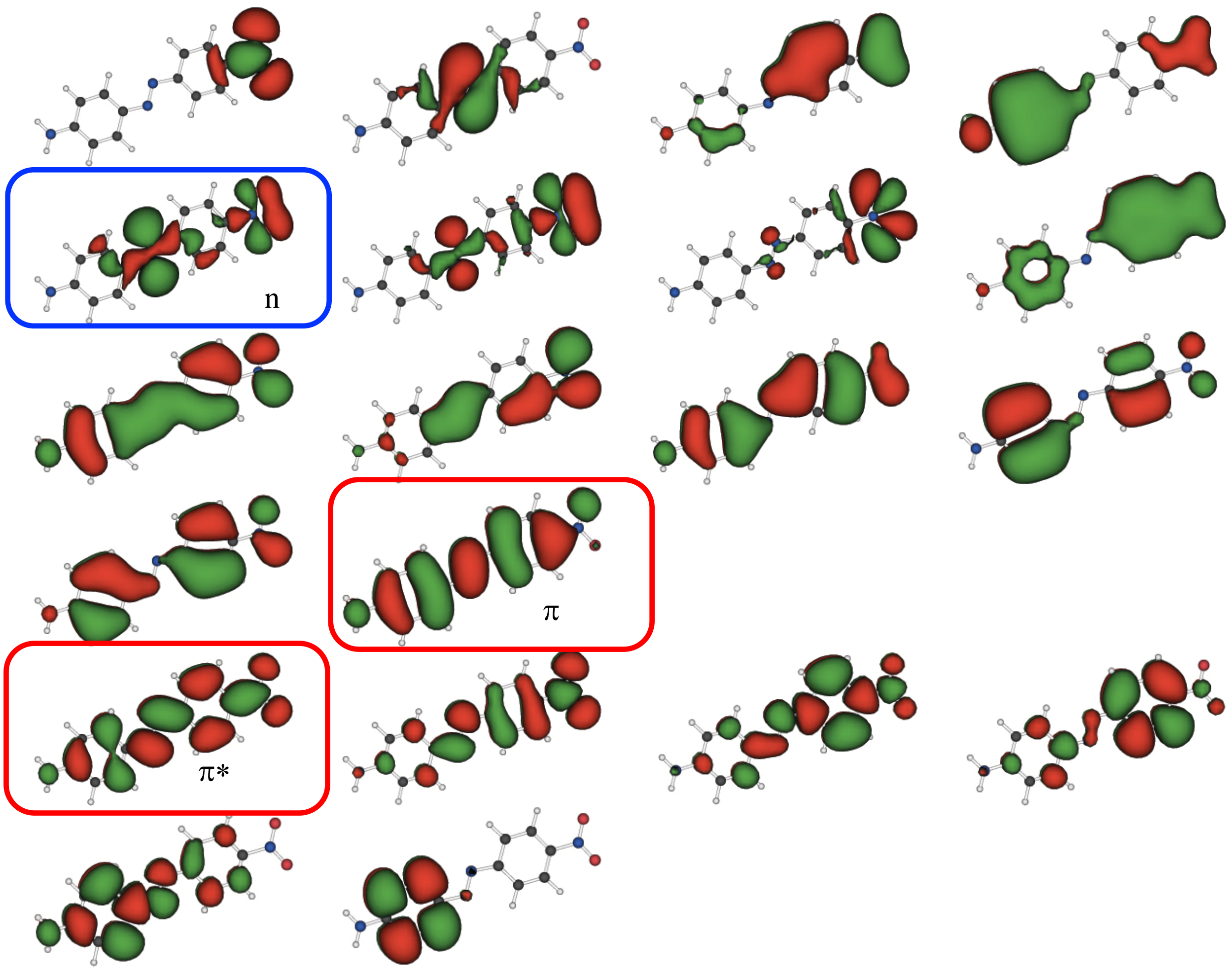

Figure S3 – Active space orbitals for *trans*-4-(4-Nitrophenylazo)aniline. Corresponding *cis*-orbitals were used with in the case of the *cis* isomer.

3. Vertical excitation energies and dipole moments of AB-*push-pull* derivatives

Table S1 – S_0 dipole moment and vertical excitation energies for azobenzene (1) and 8 *push-pull* derivatives in the gas phase (CAM-B3LYP/6-31G*)

Structure	S_0 Dipole moment (Debye)	nπ* (eV)	ππ* (eV)
1) 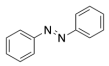	0.00	2.72 (*f*=0.00)	4.08 (*f*=0.82)
2) 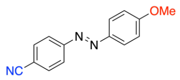	11.29	2.70 (*f*=0.00)	3.67 (*f*=1.10)
3) 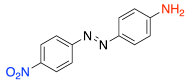	15.91	2.70 (*f*=0.00)	3.46 (*f*=1.10)
4) 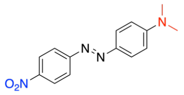	18.33	2.69 (*f*=0.00)	3.28 (*f*=1.20)
5) 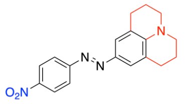	19.42	2.70 (*f*=0.00)	3.13 (*f*=1.19)
6) 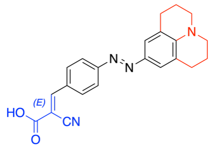	22.92	2.68 (*f*=0.00)	2.95 (*f*=1.60)
7) 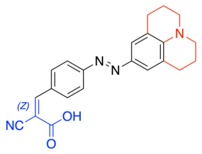	20.31	2.66 (*f*=0.00)	2.92 (*f*=1.60)
8) 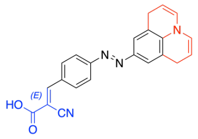	21.25	2.61 (*f*=0.00)	2.69 (*f*=0.86)
9) 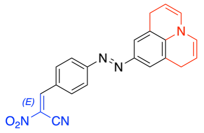	12.37	2.57 (*f*=0.00)	2.55 (*f*=0.86)

4. Excited state nature

While the first excited state is clearly identified with the nπ* excitation in all the *trans*- and *cis*-systems (dark state), the nature of the first bright state (S_2 at RASPT2/RASSCF and TD-DFT level) is often highly multiconfigurational and sometimes difficult to assign to a unique excitation. In all of the cases, however, the lowest ππ* excitation (see Figures S1, S2 and S3 for the involved orbitals) plays a major role in the description of the S_2 state, as demonstrated by the intensity of the oscillator strength. Only in the case of the *cis*- substituted systems, due to the red-shift of ππ* state and to the non-planar structure, we observe a mixing of the n and π orbitals, but still the bright state can be identified by the intensity of the transition dipole moment.

Table S2 –SA-8-RASSCF/ANO-L-VDZP weight of the ππ* and nπ* excitations in the S_1 and S_2 wavefunctions. (The nature of the orbitals n, π and π* involved in the description of the states is shown in Figure S1,Figure S2Figure S3)

		trans				
		**S_1**			**S_2**	
	**nπ***	**ππ***	**other ππ***	**nπ***	**ππ***	**other ππ***
AB	0.72	<0.005	-	-	0.57	-
NC-AB-OMe	0.72	-	-	-	0.53	-
NH_2-AB-NO_2	0.52	-	0.16	-	0.58	0.11
		cis				
		**S_1**			**S_2**	
	**nπ***	**ππ***	**other ππ***	**nπ***	**ππ***	**other ππ***
AB	0.48	0.21	-	0.11	0.27	-
NC-AB-OMe	0.56	-	-	-	0.34	-
NH_2-AB-NO_2	0.36	0.24	-	-	0.10	0.21

Table S3 – CAM-B3LYP/6-31G* weight of the ππ* and nπ* excitations in the S_1 and S_2 TD-DFT wavefunctions

		trans				
		**S_1**			**S_2**	
	**nπ***	**ππ***	**other ππ***	**nπ***	**ππ***	**other ππ***
AB	0.48	-	-	-	0.49	-
NC-AB-OMe	0.46	-	-	-	0.48	-
NH_2-AB-NO_2	0.40	-	-	-	0.45	-
		cis				
		**S_1**			**S_2**	
	**nπ***	**ππ***	**other ππ***	**nπ***	**ππ***	**other ππ***
AB	0.40	-	-	-	0.29	-
NC-AB-OMe	0.31	-	-	0.15	0.22	-
NH_2-AB-NO_2	0.10	0.18	0.10	0.12	0.26	-

5. Ground state and excited state charge distribution in the FC geometry.

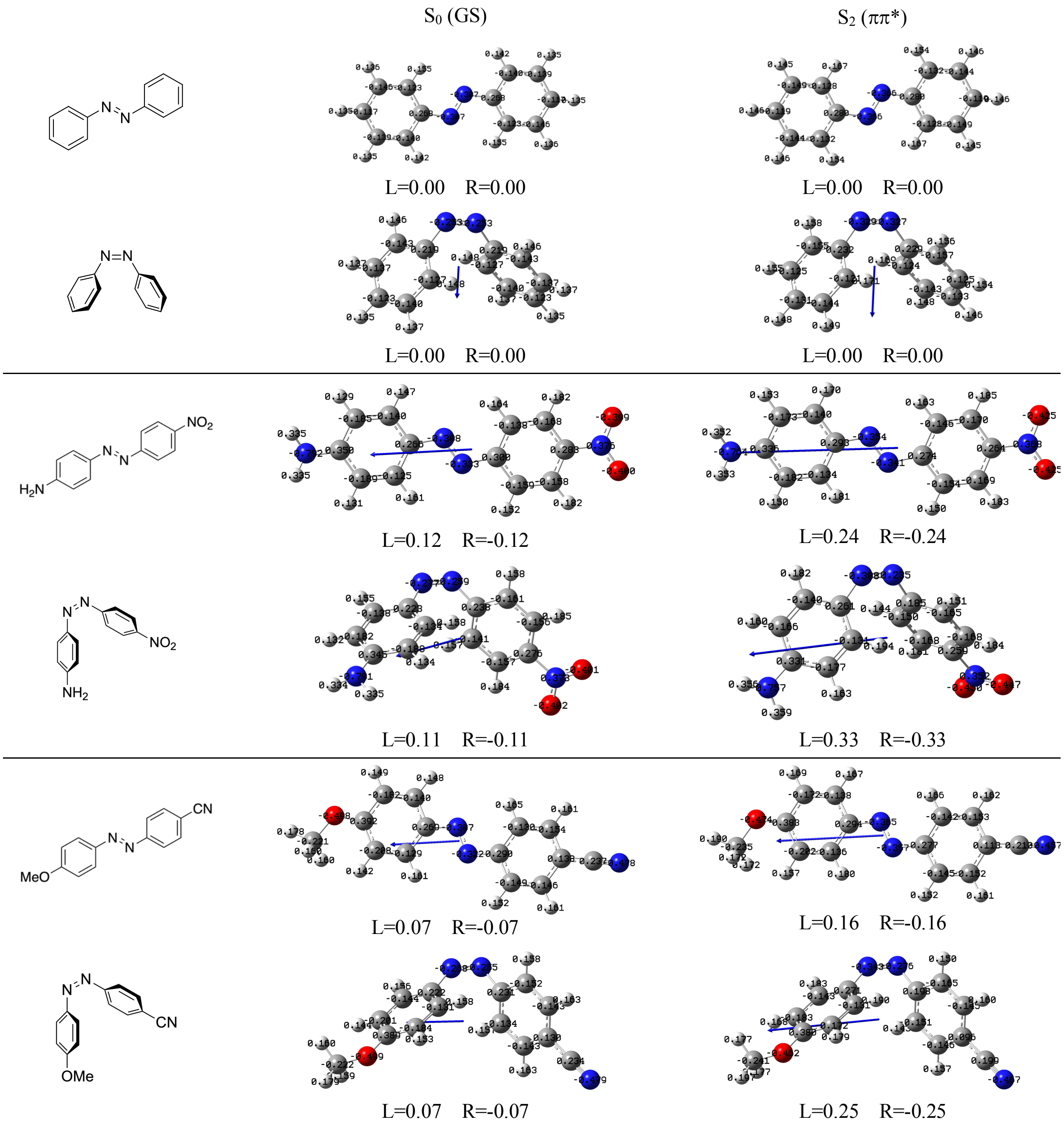

Figure S4 – Charge distribution in the ground and bright excited state for the six considered systems in the gas phase. The labels “L” and “R” below each structure indicate the total charge on the left and right half of the reported structure, respectively.

6. TD-DFT Dynamics

Table S4 - ππ* *trans*-AB trajectories parameters. Grey background for *torsional* path. CNN = largest angle, NNC = smallest angle. The average lifetimes at the bottom refer to the lifetimes on S_2 until the crossing with S_1 and to the total S_2+S_1 lifetime, before decaying on the ground state. The values in parentheses refers to the average lifetime just on S_1.

	$S_2 \rightarrow S_1$ (fs)	$S_1 \rightarrow S_0$ (fs)	Geometry at $S_2 \rightarrow S_1$ hop				Geometry at $S_1 \rightarrow S_0$ hop			
			CNNC (°)	CNN (°)	NNC (°)	NN (Å)	CNNC (°)	CNN (°)	NNC (°)	NN (Å)
1	465	637	179.83	105.16	102.71	1.37	123.79	137.38	133.80	1.33
2	380	452	173.32	107.42	104.11	1.43	121.50	149.53	140.10	1.14
3	33	57	159.30	107.39	105.37	1.44	134.07	138.52	135.82	1.33
4	60	128	179.35	107.11	105.26	1.37	130.97	142.94	139.01	1.22
5	9	23	178.46	111.88	108.62	1.44	176.75	149.61	141.57	1.28
6	185	281	177.43	108.85	107.39	1.39	172.47	150.90	144.87	1.20
7	40	55	177.62	107.66	104.73	1.45	158.42	154.84	143.35	1.23
8	775	834	172.92	107.83	106.24	1.35	145.70	145.60	140.81	1.33
9	45	132	174.96	108.19	103.49	1.43	131.39	141.24	141.17	1.22
10	44	148	173.41	107.08	105.62	1.41	163.99	161.43	152.43	1.19
11	105	172	175.44	111.95	105.41	1.47	142.21	141.09	139.56	1.33
12	86	149	177.03	106.40	106.34	1.35	179.33	146.84	145.99	1.28
13	100	162	178.24	109.53	103.63	1.36	167.92	145.77	143.66	1.23
14	150	169	165.45	109.08	104.34	1.44	149.25	150.13	137.91	1.31
15	174	281	175.49	106.93	101.83	1.43	170.51	150.76	141.14	1.23
16	842	909	178.28	105.53	104.07	1.40	169.08	149.80	146.05	1.21
17	31	63	161.24	105.04	103.58	1.30	161.70	143.64	142.80	1.19
18	36	99	165.38	110.32	110.00	1.38	109.51	164.59	158.61	1.22
19	241	258	171.13	105.68	105.20	1.45	162.17	145.23	144.65	1.31
20	36	103	177.53	111.43	111.17	1.45	139.05	143.21	137.85	1.21
21	387	411	176.48	109.98	106.91	1.38	153.75	141.77	137.87	1.23
22	211	313	179.16	107.12	101.57	1.40	148.30	147.83	137.79	1.32
23	30	102	172.83	107.57	103.26	1.36	142.98	145.03	137.77	1.30
24	12	30	178.25	109.66	104.02	1.39	169.38	147.26	143.28	1.27
25	185	244	177.11	105.72	105.49	1.35	141.05	145.73	138.33	1.27
26	84	107	174.53	110.30	102.02	1.39	153.84	147.03	142.08	1.20
27	32	56	174.58	110.47	95.17	1.29	166.48	153.91	140.98	1.23
28	67	131	171.18	106.61	104.59	1.39	168.21	154.99	142.70	1.26
29	211	274	178.82	107.95	99.18	1.44	137.08	146.76	144.41	1.26
30	33	58	177.54	102.17	100.17	1.26	165.78	165.55	154.58	1.14
31	33	92	171.19	108.94	104.91	1.35	150.82	150.81	135.99	1.32
32	64	156	177.82	105.81	103.09	1.40	154.00	149.51	146.46	1.21
33	230	284	176.03	113.91	106.75	1.49	147.23	146.13	144.63	1.13
34	363	455	179.58	111.15	105.53	1.45	144.99	144.58	143.70	1.17
35	59	122	177.30	107.64	105.42	1.46	160.35	148.57	142.79	1.30
36	628	756	177.54	107.70	103.98	1.35	129.21	141.11	135.79	1.21
37	67	137	168.36	103.98	101.79	1.36	166.24	149.83	146.45	1.19
38	11	70	178.56	113.11	112.16	1.51	150.75	144.97	144.54	1.25
39	90	146	179.21	110.55	104.75	1.43	163.63	151.88	138.57	1.26
40	68	173	173.38	102.54	101.69	1.32	135.71	142.53	134.93	1.25
average	168	231(63)	175	108	105	1.40	140	151	142	1.24
average (*torsion*)	235	323(88)	173	108	105	1.40	126	145	141	1.24
average (*bending*)	153	211(58)	175	108	104	1.40	157	149	142	1.24

Table S5 - ππ* *trans*-NC-AB-OMe trajectories parameters. Grey background for *torsional* path. The values in parentheses at the bottom of the table the average angle values of the largest and the smallest CNN angle of each $S_1 \rightarrow S_0$ hop geometry. The average lifetimes at the bottom refer to the lifetimes on S_2 until the crossing with S_1 and to the total S_2+S_1 lifetime, before decaying on the ground state. The values in parentheses refers to the average lifetime just on S_1.

	$S_2 \rightarrow S_1$ (fs)	$S_1 \rightarrow S_0$ (fs)	Geometry at $S_2 \rightarrow S_1$ hop				Geometry at $S_1 \rightarrow S_0$ hop			
			CNNC (°)	NC-CNN (°)	OMe-NNC (°)	NN (Å)	CNNC (°)	NC-CNN (°)	OMe-NNC (°)	NN (Å)
1	133	155	175.87	109.68	109.81	1.33	161.83	141.24	147.82	1.21
2	73	129	179.22	99.31	108.24	1.32	145.48	143.94	148.32	1.22
3	13	262	174.85	112.44	111.98	1.46	132.61	138.44	133.52	1.33
4	66	204	179.22	108.88	113.13	1.36	163.68	144.03	149.04	1.23
5	32	413	178.65	111.49	111.88	1.43	125.81	128.51	133.92	1.31
6	199	299	169.76	111.54	106.00	1.44	135.43	146.81	141.77	1.16
7	32	49	176.84	110.53	108.85	1.36	175.46	147.84	143.12	1.22
8	230	330	179.12	110.08	110.42	1.39	164.26	147.47	143.83	1.29
9	34	472	174.56	115.42	105.54	1.41	112.10	127.74	128.32	1.23
10	45	58	179.46	109.40	108.96	1.31	176.00	148.58	138.35	1.25
11	97	418	179.52	111.95	104.82	1.41	117.37	133.54	139.41	1.22
12	105	701	179.20	106.86	110.38	1.35	129.86	138.54	139.26	1.20
13	68	86	176.06	108.32	105.44	1.34	179.91	140.27	147.06	1.21
14	36	98	177.83	110.93	109.73	1.42	145.78	134.40	141.31	1.25
15	93	190	172.60	112.44	108.65	1.35	178.93	153.20	152.22	1.28
16	5	294	178.60	109.57	109.12	1.36	126.54	131.17	141.61	1.32
17	40	92	174.74	111.93	108.44	1.37	161.94	150.01	136.33	1.20
18	61	119	176.11	108.84	106.49	1.33	161.25	139.76	144.58	1.26
19	63	171	173.21	108.71	102.11	1.32	145.77	153.20	148.40	1.23
20	6	72	175.81	107.27	107.19	1.40	142.42	139.84	140.20	1.32
21	70	347	173.08	104.41	109.49	1.33	118.17	126.49	127.47	1.30
22	7	33	177.63	107.18	109.90	1.39	156.03	146.01	136.82	1.35
23	82	180	178.32	110.46	102.67	1.40	149.12	144.90	148.98	1.21
24	63	98	174.32	107.22	105.44	1.42	129.46	132.85	136.87	1.18
25	86	326	177.22	105.23	109.13	1.33	112.72	127.27	134.48	1.23
26	235	450	177.60	110.26	101.61	1.31	124.27	135.58	124.06	1.31
27	134	763	179.36	114.40	108.34	1.38	116.78	139.08	129.59	1.23
28	17	111	178.63	112.20	105.82	1.52	176.17	150.04	146.38	1.20
29	62	168	174.31	106.74	108.21	1.36	158.63	143.35	145.20	1.26
30	62	164	179.30	106.95	111.35	1.34	143.77	134.96	146.62	1.18
31	51	146	177.93	111.16	110.94	1.38	150.78	147.26	142.84	1.29
32	46	251	179.83	111.54	109.88	1.39	128.54	126.21	145.05	1.31
33	29	94	178.79	109.19	110.86	1.33	151.69	136.53	142.43	1.25
34	30	55	176.16	108.68	108.54	1.36	166.31	146.28	145.13	1.17
35	65	231	177.68	105.16	110.69	1.35	176.84	139.57	146.95	1.20
36	33	54	175.00	108.40	106.10	1.37	179.98	148.21	145.52	1.18
37	30	123	175.22	114.73	112.45	1.45	115.33	128.82	130.58	1.48
38	108	199	175.94	109.86	111.97	1.36	178.59	150.73	142.79	1.23
39	63	93	172.69	109.70	105.77	1.25	163.57	139.81	145.17	1.15
40	103	491	179.41	111.70	109.46	1.39	119.82	126.97	133.43	1.20
average	70	225 (155)	177	110 (111)	108 (107)	1.37	147	140 (143)	141 (137)	1.25
average (*torsion*)	75	386 (311)	177	110 (111)	108 (108)	1.39	122	132 (136)	134 (130)	1.28
average (*bending*)	67	138 (71)	176	109 (110)	108 (107)	1.36	161	145 (147)	145 (142)	1.23

Table S6 -ππ* *trans*-NO_2-AB-NH_2 trajectories parameters. Grey background for *torsional paths*. The values in parentheses at the bottom of the table the average angle values of the largest and the smallest CNN angle of each $S_1 \rightarrow S_0$ hop geometry. The average lifetimes at the bottom refer to the lifetimes on S_2 until the crossing with S_1 and to the total S_2+S_1 lifetime, before decaying on the ground state. The values in parentheses refers to the average lifetime just on S_1.

	$S_2 \rightarrow S_1$ (fs)	$S_1 \rightarrow S_0$ (fs)	Geometry at $S_2 \rightarrow S_1$ hop				Geometry at $S_1 \rightarrow S_0$ hop			
			CNNC (°)	NO_2-CNN (°)	NH_2-NNC (°)	NN (Å)	CNNC (°)	NO_2-CNN (°)	NH_2-NNC (°)	NN (A)
1	48	438	179.40	120.06	120.67	1.21	137.23	130.83	152.60	1.24
2	18	24	177.36	120.47	124.62	1.27	176.89	115.93	120.43	1.18
3	243	335	173.69	111.32	112.42	1.41	144.08	147.90	137.29	1.19
4	233	284	160.97	114.85	118.60	1.19	133.95	132.60	133.80	1.30
5	0	55	173.92	106.65	111.72	1.28	123.25	137.74	138.58	1.27
6	252	615	174.87	113.99	116.57	1.27	113.50	136.39	127.03	1.25
7	1	283	176.60	108.82	112.18	1.33	131.30	135.07	136.60	1.20
8	32	47	171.15	106.55	111.84	1.33	167.93	145.57	138.62	1.19
9	0	55	163.93	111.80	112.89	1.34	163.06	150.27	152.30	1.37
10	43	245	173.49	109.39	109.83	1.34	120.99	133.36	134.60	1.26
11	155	223	172.76	109.83	107.22	1.30	147.32	134.88	143.32	1.16
12	45	145	174.85	111.62	112.01	1.29	122.11	129.45	147.95	1.28
13	35	123	175.28	109.88	104.96	1.32	155.95	145.13	145.36	1.24
14	33	54	169.79	109.12	103.15	1.27	164.91	148.22	139.43	1.13
15	29	85	175.38	111.72	106.54	1.44	120.55	131.17	128.39	1.31
16	254	383	167.57	115.19	106.14	1.32	133.55	149.00	141.43	1.35
17	274	289	177.69	112.87	114.39	1.30	160.65	143.59	146.17	1.23
18	103	128	172.36	116.39	110.77	1.27	122.36	128.54	134.15	1.28
19	90	311	176.85	107.00	115.39	1.44	172.18	144.76	140.15	1.17
20	14	22	165.43	117.78	118.70	1.29	166.51	121.01	119.91	1.20
21	127	187	170.76	112.72	108.45	1.32	159.26	143.97	147.10	1.24
22	30	84	178.21	114.84	109.64	1.29	137.57	149.85	143.00	1.32
23	24	490	176.05	115.75	121.40	1.19	111.56	139.47	122.45	1.27
24	73	162	165.30	105.74	113.79	1.30	129.43	138.07	146.03	1.25
25	100	157	178.86	112.71	113.48	1.34	153.82	142.25	144.13	1.25
26	11	682	176.84	113.15	110.82	1.50	117.46	120.61	130.77	1.27
27	68	159	170.05	110.12	110.38	1.38	145.76	145.34	148.76	1.19
28	219	285	175.46	104.95	109.55	1.32	160.93	140.09	138.96	1.21
29	9	75	172.63	108.73	109.22	1.37	135.92	143.99	138.12	1.31
30	6	69	179.29	110.48	111.13	1.33	172.21	145.00	141.23	1.30
31	29	210	172.05	111.61	107.91	1.32	174.01	147.20	135.72	1.13
32	38	332	173.45	112.84	108.62	1.43	113.59	123.74	136.74	1.35
33	95	154	176.34	116.29	107.47	1.38	160.35	145.62	137.36	1.30
34	93	123	172.09	106.25	110.12	1.34	152.54	142.73	139.81	1.18
35	97	155	170.84	114.38	107.15	1.39	165.24	149.12	140.62	1.29
36	101	485	175.91	116.16	116.70	1.33	149.93	145.34	138.11	1.20
37	108	166	177.98	108.52	106.86	1.27	144.44	140.46	143.12	1.19
38	22	308	177.54	109.34	103.59	1.27	122.35	135.44	131.85	1.27
39	64	207	163.75	112.61	107.81	1.28	178.92	143.73	146.29	1.18
40	37	252	169.51	113.82	106.46	1.40	151.92	145.43	139.03	1.27
average	86	227(141)	173	112 (113)	111 (110)	1.32	145	139 (142)	139 (136)	1.24
average (*torsion*)	87	300(213)	173	112 (114)	112 (110)	1.32	123	134 (138)	135 (131)	1.28
average (*bending*)	85	180(95)	173	112 (113)	111 (110)	1.32	158	142 (144)	141 (139)	1.23

Table S7 - ππ* *cis*-AB trajectories parameters. CNN = largest angle, NNC = smallest angle.

	$S_2 \to S_1$ (fs)	$S_1 \to S_0$ (fs)	Geometry at $S_2 \to S_1$ hop				Geometry at $S_1 \to S_0$ hop			
			CNNC (°)	CNN (°)	NNC (°)	NN (Å)	CNNC (°)	CNN (°)	NNC (°)	NN (Å)
1	50	81	16.33	131.21	106.15	1.36	73.20	123.10	122.19	1.25
2	67	98	6.53	122.89	111.64	1.43	69.69	125.75	125.30	1.23
3	111	147	7.48	127.96	106.74	1.52	74.37	131.92	114.93	1.16
4	204	382	6.27	128.07	113.48	1.40	109.50	128.01	124.87	1.36
5	137	158	18.83	124.78	109.57	1.48	75.01	121.69	101.35	1.39
6	247	279	5.45	133.72	112.66	1.43	96.09	144.73	110.18	1.18
7	275	305	2.93	130.17	113.99	1.45	73.81	133.32	122.34	1.17
8	132	150	5.29	128.07	112.98	1.46	79.30	110.35	101.47	1.44
9	206	254	0.06	128.00	112.88	1.38	77.42	135.46	115.26	1.23
10	82	120	15.20	125.88	117.88	1.43	45.03	160.31	111.94	1.39
11	280	321	10.46	126.99	114.39	1.41	74.72	136.11	109.01	1.34
12	353	410	4.82	131.10	113.38	1.39	107.24	133.28	97.64	1.40
13	325	359	7.97	128.32	111.65	1.37	100.08	133.10	93.48	1.28
14	111	129	33.59	115.42	115.00	1.45	78.05	133.51	98.13	1.23
15	14	49	15.83	123.95	110.05	1.46	93.91	126.07	99.59	1.19
16	243	272	18.25	129.63	104.50	1.41	66.99	138.08	100.02	1.41
17	286	317	4.87	130.14	120.27	1.42	72.03	132.30	130.19	1.17
18	285	302	27.76	120.28	115.60	1.42	70.76	126.52	111.16	1.32
19	204	226	1.14	126.96	109.45	1.38	73.65	138.29	100.76	1.31
20	713	740	2.78	130.65	115.18	1.45	74.07	132.27	113.55	1.38
21	215	239	2.88	134.72	111.39	1.34	73.51	121.18	118.90	1.30
22	233	291	0.99	127.77	113.90	1.39	98.00	128.73	125.52	1.13
23	285	313	1.37	129.68	113.55	1.43	71.16	136.38	114.99	1.32
24	12	35	10.47	121.85	109.37	1.52	69.63	136.31	101.46	1.49
25	348	380	16.07	124.11	114.28	1.42	98.71	125.42	109.88	1.27
26	379	403	10.05	126.42	117.12	1.44	77.23	144.08	113.18	1.32
27	234	250	3.48	127.58	112.50	1.41	80.67	142.53	98.50	1.31
28	207	230	5.97	129.78	110.12	1.38	78.04	119.16	107.08	1.36
29	294	322	0.82	130.97	111.83	1.38	77.21	132.83	118.39	1.16
30	337	429	24.72	131.13	117.27	1.43	91.68	144.98	119.03	1.23
31	276	325	21.72	129.07	115.98	1.44	75.43	136.79	113.38	1.26
32	794	837	30.26	119.98	118.83	1.41	101.39	130.76	110.30	1.37
33	143	168	14.44	127.41	111.75	1.46	80.59	124.30	115.15	1.49
34	48	88	10.23	125.28	108.02	1.46	75.48	119.04	116.83	1.29
35	13	28	20.89	117.39	114.94	1.57	70.98	125.80	112.89	1.20
36	671	690	20.65	125.39	117.36	1.48	64.29	142.22	91.63	1.36
37	180	206	15.86	130.08	110.59	1.40	73.58	126.89	118.79	1.25
38	288	344	4.30	136.56	115.70	1.39	71.52	138.02	103.24	1.41
39	313	331	32.23	125.76	117.60	1.50	71.54	143.69	97.45	1.41
40	86	107	15.20	129.76	108.65	1.42	76.39	127.32	113.63	1.49
average	242	278	12	127	113	1.43	79	132	111	1.31

Table S8 -ππ* cis-NC-AB-OMe trajectories parameters. For the largest part of the trajectories the NNC(OMe) angle is larger than CNN(NC). The values in parentheses at the bottom of the table the average angle values of the largest and the smallest CNN angle of each $S_1 \rightarrow S_0$ hop geometry.

	$S_2 \rightarrow S_1$ (fs)	$S_1 \rightarrow S_0$ (fs)	Geometry at $S_2 \rightarrow S_1$ hop				Geometry at $S_1 \rightarrow S_0$ hop			
			CNNC (°)	CNN (°)	NNC (°)	NN (Å)	CNNC (°)	CNN (°)	NNC (°)	NN (Å)
1	120	142	13.24	134.15	108.60	1.31	76.32	110.44	139.06	1.28
2	153	182	13.63	129.27	114.69	1.41	70.12	116.29	133.59	1.21
3	9	48	0.93	127.83	109.99	1.45	78.50	121.74	122.95	1.35
4	391	461	8.03	132.87	112.57	1.35	63.40	124.93	143.20	1.20
5	157	202	1.24	131.67	112.30	1.44	92.53	135.91	117.01	1.27
6	112	132	34.04	129.01	107.23	1.38	78.09	123.78	103.14	1.39
7	53	81	17.92	136.62	107.86	1.31	74.50	129.70	111.90	1.29
8	35	93	3.78	131.98	119.65	1.48	80.80	129.79	125.38	1.36
9	139	167	0.90	129.46	112.86	1.35	73.50	105.76	125.86	1.38
10	143	174	8.50	129.52	108.54	1.37	80.91	101.33	151.40	1.28
11	182	219	3.57	130.61	112.45	1.31	75.83	126.02	133.50	1.35
12	129	177	12.38	126.99	118.57	1.46	98.15	109.79	126.81	1.35
13	80	108	20.49	133.13	106.01	1.45	54.39	104.61	147.97	1.36
14	438	481	46.44	120.47	116.09	1.40	65.64	123.21	140.81	1.32
15	265	298	12.58	135.84	112.55	1.31	68.42	114.43	158.99	1.32
16	250	365	8.02	132.90	113.55	1.38	63.46	126.13	149.31	1.27
17	344	394	12.98	129.22	109.06	1.32	67.61	121.61	145.56	1.19
18	113	150	19.55	128.71	112.38	1.40	70.06	116.97	126.94	1.39
19	172	204	18.78	132.92	113.07	1.36	72.86	108.97	142.69	1.23
20	192	213	9.22	135.53	107.46	1.37	40.41	102.82	155.65	1.37
21	173	206	22.20	130.88	107.99	1.32	102.48	106.14	134.28	1.28
22	110	132	13.12	128.88	117.06	1.45	68.72	95.76	158.93	1.37
23	145	173	1.51	132.19	115.01	1.39	97.05	107.59	134.65	1.15
24	149	193	6.83	131.15	106.41	1.36	54.04	119.43	141.47	1.45
25	73	94	2.17	134.66	110.92	1.45	80.00	120.90	112.87	1.48
26	155	177	27.51	135.36	110.91	1.40	75.28	119.91	119.54	1.43
27	227	310	17.92	135.56	113.55	1.38	68.18	126.49	142.33	1.28
28	223	264	10.61	135.95	113.06	1.37	40.11	116.71	147.48	1.39
29	120	149	4.14	131.75	112.59	1.39	70.33	109.19	139.66	1.20
30	582	606	3.65	129.49	117.76	1.36	90.75	107.87	157.39	1.27
31	229	342	7.93	136.69	113.04	1.37	106.03	130.65	126.55	1.28
32	285	367	26.09	134.43	109.14	1.40	65.89	125.41	145.67	1.26
33	234	259	7.48	132.69	114.30	1.36	74.17	117.93	125.40	1.31
34	40	59	19.93	118.98	129.55	1.57	77.06	108.66	136.95	1.41
35	120	139	28.00	123.14	114.66	1.38	78.16	123.36	109.33	1.39
36	231	292	5.58	135.20	112.96	1.38	73.37	121.65	146.28	1.23
37	189	228	37.32	121.67	110.37	1.38	61.22	121.46	138.01	1.33
38	171	203	2.65	132.66	111.96	1.39	81.58	109.49	151.43	1.20
39	165	179	41.52	132.23	105.25	1.41	77.05	115.56	116.10	1.28
40	133	174	1.40	128.79	108.05	1.32	70.45	118.80	131.54	1.33
average	181	221	14	131 (131)	112 (112)	1.39	74	117 (138)	135 (115)	1.31

Table S9 - $\pi\pi^*$ *cis*-NO_2-AB-NH_2 trajectories parameters. For the largest part of the trajectories the NNC(NH_2) angle is larger than CNN(NO_2). The values in parentheses at the bottom of the table the average angle values of the largest and the smallest CNN angle of each $S_1 \rightarrow S_0$ hop geometry.

	$S_2 \rightarrow S_1$ (fs)	$S_1 \rightarrow S_0$ (fs)	Geometry at $S_2 \rightarrow S_1$ hop				Geometry at $S_1 \rightarrow S_0$ hop			
			CNNC (°)	CNN (°)	NNC (°)	NN (Å)	CNNC (°)	CNN (°)	NNC (°)	NN (A)
1	166	194	2.43	132.58	111.97	1.33	68.07	101.11	148.97	1.31
2	148	169	3.62	117.57	131.61	1.25	14.28	134.34	120.45	1.28
3	35	63	19.76	132.78	128.59	1.14	88.81	132.37	107.46	1.39
4	17	33	23.68	118.01	128.40	1.19	75.48	107.11	123.02	1.42
5	191	212	12.38	127.97	128.80	1.22	71.13	115.90	134.89	1.28
6	17	39	25.61	112.29	136.74	1.22	72.99	118.27	123.68	1.22
7	37	79	4.56	132.44	108.60	1.33	76.54	120.67	110.09	1.34
8	96	120	12.40	132.25	127.53	1.24	99.98	126.19	108.43	1.30
9	80	102	23.26	122.68	122.29	1.28	69.64	121.72	130.11	1.27
10	199	217	17.97	123.84	123.65	1.29	73.48	118.31	114.72	1.36
11	29	43	15.31	135.36	121.92	1.22	75.33	122.39	130.10	1.30
12	413	432	13.89	118.23	130.88	1.26	75.77	110.65	127.56	1.30
13	250	263	16.48	124.68	130.19	1.24	74.92	118.97	125.73	1.29
14	209	239	12.07	119.13	134.90	1.20	90.12	131.24	122.64	1.26
15	145	184	17.44	119.10	124.69	1.23	3.58	117.60	135.09	1.27
16	20	46	23.15	130.14	122.16	1.21	75.46	107.56	123.76	1.24
17	42	58	11.64	124.92	129.17	1.18	72.44	117.14	122.63	1.27
18	56	87	12.33	124.45	125.51	1.25	76.11	112.59	121.98	1.24
19	8	34	0.70	124.11	132.58	1.22	14.12	123.78	125.19	1.22
20	152	187	2.99	117.89	133.50	1.24	98.23	110.66	115.37	1.31
21	68	86	13.10	122.05	133.56	1.19	83.14	118.82	122.82	1.25
22	30	46	11.48	125.01	131.50	1.20	79.70	122.61	128.57	1.24
23	102	116	26.70	135.22	123.82	1.29	77.90	111.70	126.13	1.32
24	55	90	1.80	124.29	120.47	1.25	89.10	118.66	116.15	1.30
25	182	209	5.38	128.14	134.17	1.31	99.96	123.06	121.37	1.25
26	104	143	1.46	132.63	114.30	1.30	70.65	111.46	143.24	1.33
27	155	172	13.90	119.87	132.02	1.30	72.20	119.39	135.22	1.32
28	36	78	29.34	129.40	130.88	1.27	77.77	124.86	130.07	1.29
29		30	4.47	123.93	124.40	1.30	75.86	115.88	131.18	1.19
30	199	239	1.32	133.41	112.99	1.35	53.71	112.99	142.63	1.39
31	61	89	12.82	136.87	106.01	1.41	74.60	125.49	124.51	1.24
32	96	115	27.24	133.88	103.98	1.37	80.97	121.45	111.68	1.40
33	89	163	5.48	118.42	130.89	1.20	101.20	111.32	126.90	1.30
34	31	53	10.07	137.88	122.21	1.15	100.85	122.60	114.91	1.23
35	167	191	9.60	125.78	117.86	1.27	68.99	104.96	147.28	1.26
36	80	103	5.72	125.39	117.20	1.24	90.10	138.31	118.17	1.23
37	80	85	75.87	116.08	109.46	1.40	78.70	111.33	118.45	1.27
38	573	607	11.57	131.46	119.72	1.33	95.94	117.18	119.75	1.25
39	183	209	14.57	131.87	116.83	1.19	77.46	116.36	127.04	1.24
40	107	130	1.74	124.55	131.38	1.25	71.16	120.95	130.11	1.28
average	118	144	14	126 (131)	124 (120)	1.26	75	118 (128)	125 (115)	1.29

Table S10 - nπ* *trans*-AB trajectories parameters. Grey background for *torsional* path.

	$S_1 \rightarrow S_0$ (fs)	Geometry at $S_1 \rightarrow S_0$ hop			
		CNNC (°)	CNN (°)	NNC (°)	NN (A)
1	176	125.99	139.58	137.15	1.23
2	63	81.03	154.78	150.96	1.22
3	24	163.06	148.88	145.73	1.21
4	135	150.35	145.75	140.32	1.23
5	19	168.18	148.78	141.41	1.26
6	17	177.89	147.96	141.42	1.20
7	56	171.09	154.14	142.46	1.27
8	182	128.77	137.80	133.24	1.18
9	16	150.37	149.44	140.53	1.23
10	539	119.36	132.76	130.76	1.22
11	232	117.40	135.02	125.86	1.33
12	725	102.39	131.15	127.86	1.26
13	59	152.13	145.37	139.58	1.18
14	58	122.64	141.47	130.02	1.26
15	20	179.33	151.05	144.41	1.22
16	24	133.64	140.82	139.39	1.20
17	63	170.94	147.66	141.91	1.15
18	409	127.90	146.28	134.38	1.22
19	20	166.07	148.76	143.63	1.18
20	58	153.08	144.36	144.21	1.21
21	57	156.90	149.62	141.87	1.20
22	98	151.91	146.26	140.78	1.20
23	177	136.30	136.80	133.68	1.24
24	19	146.63	145.78	141.32	1.23
25	20	129.79	140.40	135.41	1.23
26	284	125.31	141.67	137.91	1.28
27	51	145.85	150.95	145.90	1.28
28	91	139.04	146.62	135.06	1.28
29	56	159.32	149.33	145.19	1.18
30	64	154.16	144.00	143.97	1.19
31	95	148.75	141.31	140.10	1.26
32	20	156.92	144.99	139.16	1.25
33	534	115.90	130.27	126.14	1.23
34	55	150.89	145.72	139.56	1.30
35	70	155.77	146.20	145.23	1.29
36	175	159.53	148.68	136.44	1.26
37	31	144.73	144.77	138.04	1.24
average	130	144	144	139	1.23
average (*torsion*)	270	119	139	134	1.24
average (*bending*)	62	156	147	141	1.23

7. TD-DFT *vs* RASPT2 energies at S_1/S_0 crossings (0K dynamics)

Table S11 - Comparison between TD-DFT and RASSCF/RASPT2 energy values of the S_1/S_0 CIs, reached along the 0K dynamics starting from the *trans*-AB derivatives excited on the ππ* (S_2) state.

	TD-DFT			RASSCF/RASPT2		
	S_0 (eV)	S_1 (eV)	ΔE (eV)	S_0 (eV)	S_1 (eV)	ΔE (eV)
AB	3.17	3.25	0.08	3.74	3.80	0.06
O_2N-AB-NH_2	2.72	2.78	0.06	3.20	3.31	0.11
NC-AB-OMe	2.78	2.90	0.12	3.24	3.29	0.05

The RASPT2 calculations were here performed only for the S_1/S_0 CIs, that need more care due to the inaccuracy of TD-DFT near crossings between ESs and the GS.

Concerning the S_2/S_1 crossing, in our 0K dynamics it takes place at CNNC=180°, CNN=NNC=107°, a structure that is RASPT2-validated by past calculations published by our group[2,3].

8. Cartesian coordinates of the B3LYP/DFT/6-31G* optimized ground state minima

Trans-AB

N	-0.081666	0.000000	0.048011
N	0.081662	0.000000	1.298005
C	1.435775	0.000000	1.721203
C	-1.435776	0.000000	-0.375199
C	1.630458	0.000000	3.108770
C	2.920569	0.000000	3.636795
C	4.021744	0.000000	2.779117
C	3.829233	0.000000	1.391261
C	2.545724	0.000000	0.858384
H	0.754460	0.000000	3.750370
H	3.066267	0.000000	4.713456
H	5.029189	0.000000	3.186609
H	4.689141	0.000000	0.726455
H	2.375272	0.000000	-0.212367
C	-1.630446	0.000000	-1.762769
C	-2.920552	0.000000	-2.290807
C	-4.021735	0.000000	-1.433141
C	-3.829238	0.000000	-0.045283
C	-2.545734	0.000000	0.487607
H	-0.754442	0.000000	-2.404361
H	-3.066239	0.000000	-3.367470
H	-5.029177	0.000000	-1.840643
H	-4.689153	0.000000	0.619514
H	-2.375293	0.000000	1.558359

Cis-AB

N	0.455845	-0.009574	0.048302
N	0.454824	-0.034513	1.297490
C	1.642581	-0.000303	2.103764
C	1.636293	-0.145350	-0.758005
C	2.658759	0.950773	1.928533
C	3.699780	1.024560	2.851922
C	3.746725	0.147388	3.938600
C	2.725840	-0.789422	4.118870
C	1.661034	-0.844440	3.222169
H	2.623110	1.632799	1.085596
H	4.480071	1.769330	2.719920
H	4.565775	0.205085	4.650008
H	2.747642	-1.463073	4.971178
H	0.837547	-1.537354	3.368150
C	2.565310	-1.182100	-0.585145
C	3.596362	-1.344037	-1.508472
C	3.720594	-0.471074	-2.592438
C	2.785698	0.552045	-2.770227
C	1.729211	0.697278	-1.873794
H	2.469146	-1.860335	0.256119
H	4.307986	-2.155003	-1.378546
H	4.531805	-0.598423	-3.303766
H	2.867160	1.223799	-3.620416
H	0.969946	1.460388	-2.017931

Trans-NC-AB-OMe

C	-2.572524	0.000000	0.530140
C	-1.491840	-0.000001	-0.370707
C	-1.738010	-0.000001	-1.751378
C	-3.039593	0.000000	-2.237400
C	-4.117956	0.000001	-1.336804
C	-3.872977	0.000001	0.051372
N	-0.125774	-0.000002	0.004748
N	0.070433	-0.000002	1.253958
C	1.419249	-0.000001	1.652164
C	1.648429	-0.000001	3.039928
C	2.937550	0.000000	3.544905
C	4.032905	0.000000	2.664880
C	3.815624	0.000000	1.274316
C	2.519180	-0.000001	0.777548
H	0.788801	-0.000001	3.703068
H	3.130009	0.000000	4.612750
O	5.257044	0.000001	3.250735
H	4.650731	0.000000	0.583319
H	2.336112	-0.000001	-0.291236
H	-0.887614	-0.000002	-2.425727
H	-3.231124	0.000000	-3.305444
H	-4.712273	0.000001	0.739773
H	-2.366331	0.000000	1.594124
C	6.413735	0.000001	2.423089
H	7.265431	0.000001	3.104939
H	6.453890	-0.895543	1.790415
H	6.453889	0.895545	1.790415
C	-5.464149	0.000001	-1.827188

Cis-NC-AB-OMe

C	1.619459	0.817705	-1.951384
C	1.621215	0.065145	-0.769802
C	2.594915	-0.941286	-0.606014
C	3.554383	-1.145865	-1.582738
C	3.583023	-0.341905	-2.737141
C	2.606549	0.648016	-2.918356
N	0.509695	0.303463	0.091096
N	0.529673	0.165690	1.335238
C	1.708316	-0.035090	2.116932
C	2.773846	0.880676	2.095342
C	3.815569	0.755688	3.004610
C	3.815194	-0.291804	3.943354
C	2.738725	-1.196619	3.974277
C	1.683150	-1.051854	3.083802
H	2.774194	1.688540	1.371199
H	4.638291	1.463503	2.992989
H	2.731547	-1.995991	4.708239
H	0.830140	-1.722319	3.120813
H	2.582952	-1.579792	0.269438
H	4.299897	-1.928005	-1.480731
O	4.578605	-0.616829	-3.617945
H	2.592370	1.264392	-3.809488
H	0.831148	1.550920	-2.092668
C	4.656135	0.144618	-4.816538
H	5.521249	-0.241333	-5.357612
H	3.755784	0.017356	-5.430723
H	4.804921	1.211002	-4.604729
C	4.898380	-0.427886	4.870326

Trans-O_2N-AB-NH_2

C	-2.521145	0.012885	0.477933
C	-1.423578	-0.000133	-0.404782
C	-1.646315	-0.018820	-1.791038
C	-2.938731	-0.024196	-2.301183
C	-4.008307	-0.010867	-1.406625
C	-3.814214	0.007515	-0.021658
N	-0.065842	0.003521	-0.007184
N	0.105913	0.023294	1.248803
C	1.438128	0.026357	1.675303
C	1.639864	0.050002	3.066208
C	2.916531	0.058459	3.603651
C	4.042032	0.042090	2.755456
C	3.839442	0.017207	1.354742
C	2.564351	0.009538	0.825106
H	0.765605	0.064302	3.710005
H	3.057304	0.073746	4.681593
N	5.319651	0.000997	3.275873
H	4.703987	0.000028	0.694756
H	2.406592	-0.007194	-0.247543
H	-0.784365	-0.028972	-2.450239
H	-3.132860	-0.038433	-3.366348
N	-5.377272	-0.016300	-1.934184
H	-4.676552	0.017071	0.633749
H	-2.333470	0.026843	1.545029
O	-6.305213	-0.004518	-1.123513
O	-5.521229	-0.032245	-3.157881
H	6.078499	0.274759	2.666911
H	5.440892	0.296093	4.234994

Cis-O_2N-AB-NH_2

C	1.605427	0.565799	-2.051882
C	1.669281	-0.022717	-0.775555
C	2.747365	-0.883817	-0.487892
C	3.738971	-1.105489	-1.429107
C	3.706411	-0.461485	-2.684684
C	2.615686	0.381238	-2.980418
N	0.534690	0.224427	0.039563
N	0.525014	0.176003	1.292474
C	1.676939	0.096220	2.123803
C	2.655694	1.108008	2.110129
C	3.668380	1.109992	3.059064
C	3.704304	0.094907	4.017523
C	2.732031	-0.905939	4.061516
C	1.706039	-0.888925	3.125799
H	2.609760	1.888048	1.357420
H	4.433141	1.876970	3.068854
N	4.783977	0.085608	5.005925
H	2.786535	-1.668170	4.829022
H	0.918995	-1.635885	3.153502
H	2.796832	-1.406021	0.459816
H	4.552207	-1.791206	-1.203539
N	4.685058	-0.710517	-3.627645
H	2.558178	0.863475	-3.953145
H	0.745429	1.186391	-2.284594
O	4.792863	-0.822599	5.839208
O	5.625563	0.984500	4.946321
H	4.783795	-0.040367	-4.377968
H	5.567075	-1.073482	-3.292697

REFERENCES

(1) Du, L.; Lan, Z. An On-the-Fly Surface-Hopping Program JADE for Nonadiabatic Molecular Dynamics of Polyatomic Systems: Implementation and Applications. *J. Chem. Theory Comput.* **2015**, *11* (4), 1360–1374. https://doi.org/10.1021/ct501106d.

(2) Nenov, A.; Borrego-Varillas, R.; Oriana, A.; Ganzer, L.; Segatta, F.; Conti, I.; Segarra-Marti, J.; Omachi, J.; Dapor, M.; Taioli, S.; Manzoni, C.; Mukamel, S.; Cerullo, G.; Garavelli, M. UV-Light-Induced Vibrational Coherences: The Key to Understand Kasha Rule Violation in Trans-Azobenzene. *J. Phys. Chem. Lett.* **2018**, *9* (7), 1534–1541. https://doi.org/10.1021/acs.jpclett.8b00152.

(3) Aleotti, F.; Soprani, L.; Nenov, A.; Berardi, R.; Arcioni, A.; Zannoni, C.; Garavelli, M. Multidimensional Potential Energy Surfaces Resolved at the RASPT2 Level for Accurate Photoinduced Isomerization Dynamics of Azobenzene. *J. Chem. Theory Comput.* **2019**, *15* (12), 6813–6823. https://doi.org/10.1021/acs.jctc.9b00561.

(4) Conti, I.; Martínez-Fernández, L.; Esposito, L.; Hofinger, S.; Nenov, A.; Garavelli, M.; Improta, R. Multiple Electronic and Structural Factors Control Cyclobutane Pyrimidine Dimer and 6-4 Thymine-Thymine Photodimerization in a DNA Duplex. *Chem. - Eur. J.* **2017**, *23* (60), 15177–15188. https://doi.org/10.1002/chem.201703237.

(5) Weingart, O.; Nenov, A.; Altoè, P.; Rivalta, I.; Segarra-Martí, J.; Dokukina, I.; Garavelli, M. COBRAMM 2.0 — A Software Interface for Tailoring Molecular Electronic Structure Calculations and Running Nanoscale (QM/MM) Simulations. *J. Mol. Model.* **2018**, *24* (9), 271. https://doi.org/10.1007/s00894-018-3769-6.

(6) M. J. Frisch, G. W. Trucks, H. B. Schlegel, G. E. Scuseria, M. A. Robb, J. R. Cheeseman, G. Scalmani, V. Barone, G. A. Petersson, H. Nakatsuji, X. Li, M. Caricato, A. Marenich, J. Bloino, B. G. Janesko, R. Gomperts, B. Mennucci, H. P. Hratchian, J. V. Ortiz, A. F. Izmaylov, J. L. Sonnenberg, D. Williams-Young, F. Ding, F. Lipparini, F. Egidi, J. Goings, B. Peng, A. Petrone, T. Henderson, D. Ranasinghe, V. G. Zakrzewski, J. Gao, N. Rega, G. Zheng, W. Liang, M. Hada, M. Ehara, K. Toyota, R. Fukuda, J. Hasegawa, M. Ishida, T. Nakajima, Y. Honda, O. Kitao, H. Nakai, T. Vreven, K. Throssell, J. A. Montgomery, Jr., J. E. Peralta, F. Ogliaro, M. Bearpark, J. J. Heyd, E. Brothers, K. N. Kudin, V. N. Staroverov, T. Keith, R. Kobayashi, J. Normand, K. Raghavachari, A. Rendell, J. C. Burant, S. S. Iyengar, J. Tomasi, M. Cossi, J. M. Millam, M. Klene, C. Adamo,

R. Cammi, J. W. Ochterski, R. L. Martin, K. Morokuma, O. Farkas, J. B. Foresman, and D. J. Fox. *Gaussian 09 Revision E.01*.

(7) Galván, I. F.; Vacher, M.; Alavi, A.; Angeli, C.; Autschbach, J.; Carlson, R. K.; Chibotaru, L. F.; Creutzberg, J.; Dattani, N.; Delcey, M. G.; Dong, S. S.; Dreuw, A.; Freitag, L.; Frutos, L. M.; Gagliardi, L.; Giussani, A.; González, L.; Grell, G.; Guo, M.; Hoyer, C. E.; Johansson, M.; Knecht, S.; Kovačević, G.; Källman, E.; Manni, L.; Lundberg, M.; Ma, Y.; Mai, S.; Malhado, P.; Malmqvist, P. Å.; Marquetand, P.; Norell, J.; Olivucci, M.; Oppel, M.; Phung, Q. M.; Pierloot, K.; Plasser, F.; Sand, A. M.; Schapiro, I.; Sharma, P.; Stein, C. J.; Sørensen, L. K.; Truhlar, D. G.; Ugandi, M.; Ungur, L.; Valentini, A.; Vancoillie, S.; Veryazov, V.; Weser, O.; Widmark, P.-O.; Zobel, J. P.; Lindh, R. OpenMolcas: From Source Code to Insight. 127.
